# Locomotor activity as an effective measure of the severity of inflammatory arthritis in a mouse model

**DOI:** 10.1371/journal.pone.0291399

**Published:** 2024-01-17

**Authors:** Mélina R. Doucet, Angela M. Laevski, Jérémie A. Doiron, Luc H. Boudreau, Marc E. Surette

**Affiliations:** Department of Chemistry and Biochemistry, New Brunswick Centre for Precision Medicine, Université de Moncton, Moncton, New Brunswick, Canada; UCSI: UCSI University, MALAYSIA

## Abstract

**Objective:**

Mouse models are valuable in preclinical studies of inflammatory arthritis. However, current methods for measuring disease severity or responses to treatment are not optimal. In this study a smart cage system using multiple sensors to measure locomotor activity was evaluated in the K/BxN serum transfer model of inflammatory arthritis.

**Methods:**

Arthritis was induced in C57BL/6 mice with injections of K/BxN serum. Clinical index and ankle thickness were measured for 14 days. Locomotor activity was measured in smart cages for 23 h periods on Days 0, 7, and 13. The same measurements were taken in mice consuming diets supplemented or not with fish oil to evaluate a preventative treatment.

**Results:**

Initiation, peak and resolution phases of disease could be measured with the smart cages. Locomotor activity including speed, travel distance, number of active movements and rear movements were all significantly lower on Days 7–8 of illness (peak) compared to Days 0 and 13–14 (resolution) (one-way repeated measures analyses, p<0.05). The clinical index and ankle thickness measurements did not capture differences between dietary groups. Significantly increased activity was measured in most of the locomotor parameters in the fish oil group compared to the control mice at both Days 8 and 14 (2-way repeated measures ANOVA, p<0.05).

**Conclusion:**

The measurement of locomotor activity provided a more detailed evaluation of the impact of inflammatory arthritis on animal well-being and mobility than that provided by measuring clinical index and ankle thickness, and could be a valuable tool in preclinical studies of inflammatory arthritis.

## Introduction

Rheumatoid arthritis is an autoimmune disease resulting in chronic inflammation of the joints [1]. Current treatments for this disease include non-steroidal anti-inflammatory drugs (NSAIDs), disease-modifying anti-rheumatic drugs (DMARDs) and biologics [2, 3]. However, in some cases these can be expensive, they are not always effective and can have side effects [2, 4]. The search for new treatments for this disease therefore remains of interest to provide alternatives to patients whose current treatments are not adequate. As a result, investigation of new potential therapeutics often rely on preclinical mouse models of arthritis that develop the disease very similarly to humans [5].

In such studies using mouse models, the severity of arthritis can be measured in different ways. These include evaluating the clinical index, measuring ankle thickness, performing gait analysis like using the Rotarode or treadmill tests, using the Von Frey test, the open field activity system, weight bearing analysis and using a voluntary running wheel [6–12]. While each of these methods can yield useful data, each have limitations. The clinical index relies on subjective evaluations while the measurement of ankle thickness is often limited to the back paws. Although gait analyses also generate useful data, the methods force the mouse, which is often in pain, to walk or run until exhaustion [11, 13, 14]. These techniques also require conditioning of the animal before the start of the experiment. The use of a voluntary running wheel is an alternative to measure the level of activity in arthritic mice where one can record the number of times the wheel spins in a specified period [12, 15]. Again, however, some aspects of the disease, like morning stiffness or the level of sedentariness are not measured, and the mouse must want to run to get data. Other behavior analyses like the Von Frey test, the open field activity system and weight bearing test can also be useful to measure arthritis in mice, but these all measure the behavior of the mice at a certain period and do not consider every aspect of the disease like again, morning stiffness or sedentariness [6–9, 14, 16]. In general, such behavioral analyses are conducted outside of the animal’s cage and this can induce unnecessary stress and is not indicative of the animal’s normal daily behavior. The development of new techniques to measure arthritis in mice, considering all aspects of their behavior during the disease, is therefore desired.

Recently, pain-like behaviors were measured in rodents using an automated home-cage system for a period of 24 h in non-arthritic lipopolysaccharide (LPS) and carrageenan inflammation models [17, 18]. These studies demonstrated that locomotion, speed, rearing and the distance traveled were diminished in mice experiencing pain compared to the control group [17, 18]. Similarly, a smart cage system was developed that allows the locomotor behavior of mice to be recorded for extended periods in the animal’s cage using sensors that monitor movements [19]. These smart cages record many of the animals’ movements such as distance traveled, speed, the number of rears, left and right rotations, and have been used for assessment of neurological disease or injury [19–24]. However, the use of a smart cage system to measure locomotor activity of arthritic mice has never been evaluated. This research tool could possibly provide valuable data for studies focused on the treatment of arthritis by measuring the animal’s regular locomotor behavior and even target times of the day that are differentially affected by the disease. In the current study, the smart cage system was used to measure aspects of animal locomotor activity over time in the K/BxN serum transfer model of inflammatory arthritis [25, 26]. The results show that this cage system can be a valuable tool to measure disease progression (initiation, peak and resolution) and the impact of potential therapeutic treatments.

## Methods

### Murine models and serum transfer from K/BxN mice to C57BL/6

Non-obese diabetic (NOD) mice (The Jackson Laboratories) were crossed with KRN transgenic mice (a generous donation from Dr. Christophe Benoist, Havard Medical School) to generate the arthritic K/BxN offspring as previously described [27]. Serum was obtained from these 6-10-week-old arthritic K/BxN mice after cardiac puncture and blood clotting. Arthritis was induced in C57BL/6 male mice by intraperitoneal injections of 100 μl of K/BxN serum on Days 0 and 2 as previously described [25, 26]. Inflammatory clinical markers were clinical index and ankle swelling that were then monitored for 14 consecutive days. Paw swelling was determined by daily ankle thickness measurements (J15 Pocket Dial Thickness Gauge, Newman Tools) and clinical score evaluation (maximum score of 12 per animal, 3 per limb with the next scoring: 0, no evidence of inflammation; 1, inflammation in any of the following aspects: individual phalanx joints, localized wrist/ankle, or swelling on the surface of the paw; 2, inflammation in two aspects of the paw 3, major swelling on all aspects of the paw) [25, 26].

### Mouse behavior analysis using SmartCage^™^

The SmartCage^™^ system (smart cages) was from Afasci Research Laboratory (Redwood City, CA, USA). They provide an inner space of 36 × 23 × 9 cm (length × width × height) for placement of mouse cages with bedding, water and food as previously described [19]. Throughout the experiment, four cages at a time were connected to a computer and data were recorded with the CageCenter^™^ software. Mice were placed in the cages that were within the inner space of the smart cage from Day -1 for 23 hours prior to the first serum injections and were removed from the smart cages on Day 0. Mice were again placed in smart cages for 23 hours from Day 7 to 8 (Day 8 readings), and from Day 13 to Day 14 (Day 14 readings) (n = 6–8 mice). Data were continuously collected by the multiple sensors in the smart cages. An event was tabulated when a sensor was triggered. Data were reported as number of active movements, horizontal movements and rear movements; minutes of active time, time moving horizontally, rearing time and time spent performing fine movements; percentage of time active, of time rearing, and of time moving horizontally; total distance traveled and fine movements distance; average speed (cm/s); number of left-rotations, number of right-rotations and net rotations [19]. Rotations were calculated when the animal made a 360° turn and rearing was measured when the top sensors of the cage were triggered. Data were analysed with the CageScore^™^ Windows-based program.

### Dietary intervention study

Animals were divided into two dietary groups. One group was provided with a regular chow diet (diet D12450HN, Research Diets, New Brunswick, NJ) while the second group was provided with the same chow diet in which 80% of the dietary lipids were replaced with fish oil (Fish oil diet). The experimental diets were isocaloric and the fatty acid composition of the diets is shown in S1 Table. Water and food were provided ad libitum, fresh food was provided as needed and uneaten food was discarded to minimize oxidation. Animals consumed the experimental diets for 3 weeks prior to the induction of arthritis and continued to consume their respective diets throughout the study. The investigators were blinded to the dietary groups until data analysis was completed.

### Ethics

All protocols were approved by the Université de Moncton Animal protection committee (protocols 19–08 and 19–09) which follows the guidelines of the Canadian Council on Animal Care. Animals were monitored daily throughout the study for signs of distress such as loss of appetite, trembling, loss of coordination, hunched posture, or poor coat condition. The protocol dictated that such animals in distress be sacrificed to alleviate suffering. At the end of the study animals were sedated by inhalation of 2–3% isoflurane and were then sacrificed by cervical dislocation.

### Statistical analysis

Statistical analyses were performed to test two hypotheses. The first hypothesis was that smart cages can be used to detect changes in locomotor activity over the course of disease progression in arthritic mice. The second hypothesis was that smart cages can be used to detect differences in locomotor activity between arthritic mice consuming a control chow diet and arthritic mice consuming a diet enriched in fish oil at day 8 (peak phase) and day 14 (resolution phase). Data are presented as means +/- SD and analyzed with GraphPad Prism software (version 9.4.1, GraphPad Software Inc.). One-way and two-way repeated measures ANOVA tests were performed, with Tukey or Šídák’s multiple comparison analyses as indicated in the figure legends. In cases where there were missing data points due to loss of data, data were analysed as repeated measures by fitting a mixed model. In one instance, a two-tailed t-test was used to compare areas under the curve values. In all cases, differences were deemed significant if p-values were <0.05.

## Results

### Analysis of the progression of arthritis with smart cages

**Fig 1:**
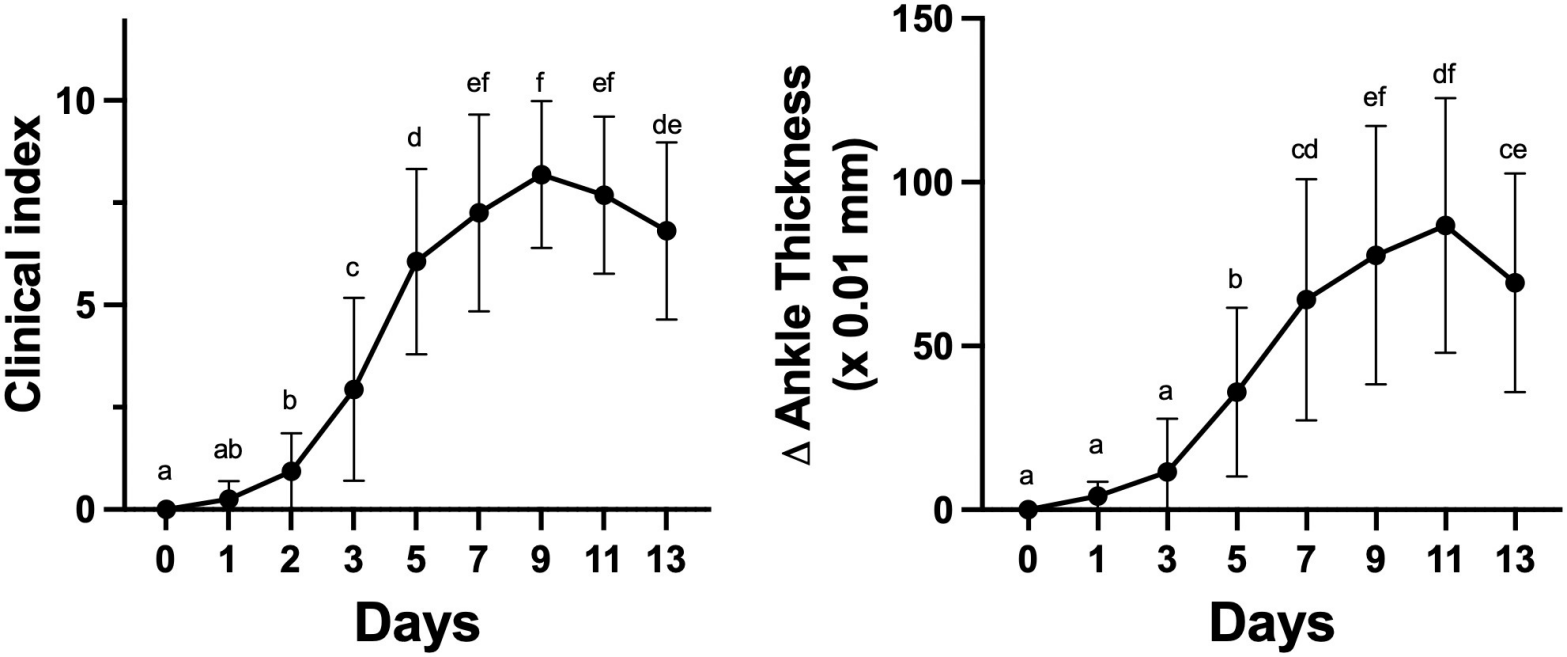
Ankle thickness and clinical index measurements over time. Arthritis was induced with injections of arthritogenic K/BxN serum into C57BL/6 mice on days 0 and 2. Change in clinical index and change in ankle thickness of mouse joints were then measured over time as described in the Methods. One-way repeated measures ANOVA were conducted. Multiple comparisons were performed using the Tukey multiple comparison test. Values without at least one common superscript letter were different (p<0.05). Values are means +/- SD, n = 14 animals for ankle thickness measurements and n = 16 for clinical index measurements.

Arthritis was induced in C57BL/6 mice by injecting (i.p.) 100 μl of KBx/N serum on Days 0 and 2 of the experiment. Ankle thickness and clinical index were monitored for 14 days. Repeated measures one-way ANOVA analyses showed that clinical index (F (2.727, 40.91) = 119.8, p<0.001) and changes in ankle thickness (F (2.228, 28.96) = 39.97, p<0.001) changed significantly over time. The results in Fig 1 demonstrate that the inflammation progressed until Day 9 reaching a maximum between Days 9 and 11. Both clinical index and ankle thickness scores then showed a decrease by Day 13 which could be considered the beginning of the resolution phase of inflammation. Overall, these results confirm the development of inflammatory arthritis in these animals.

**Fig 2:**
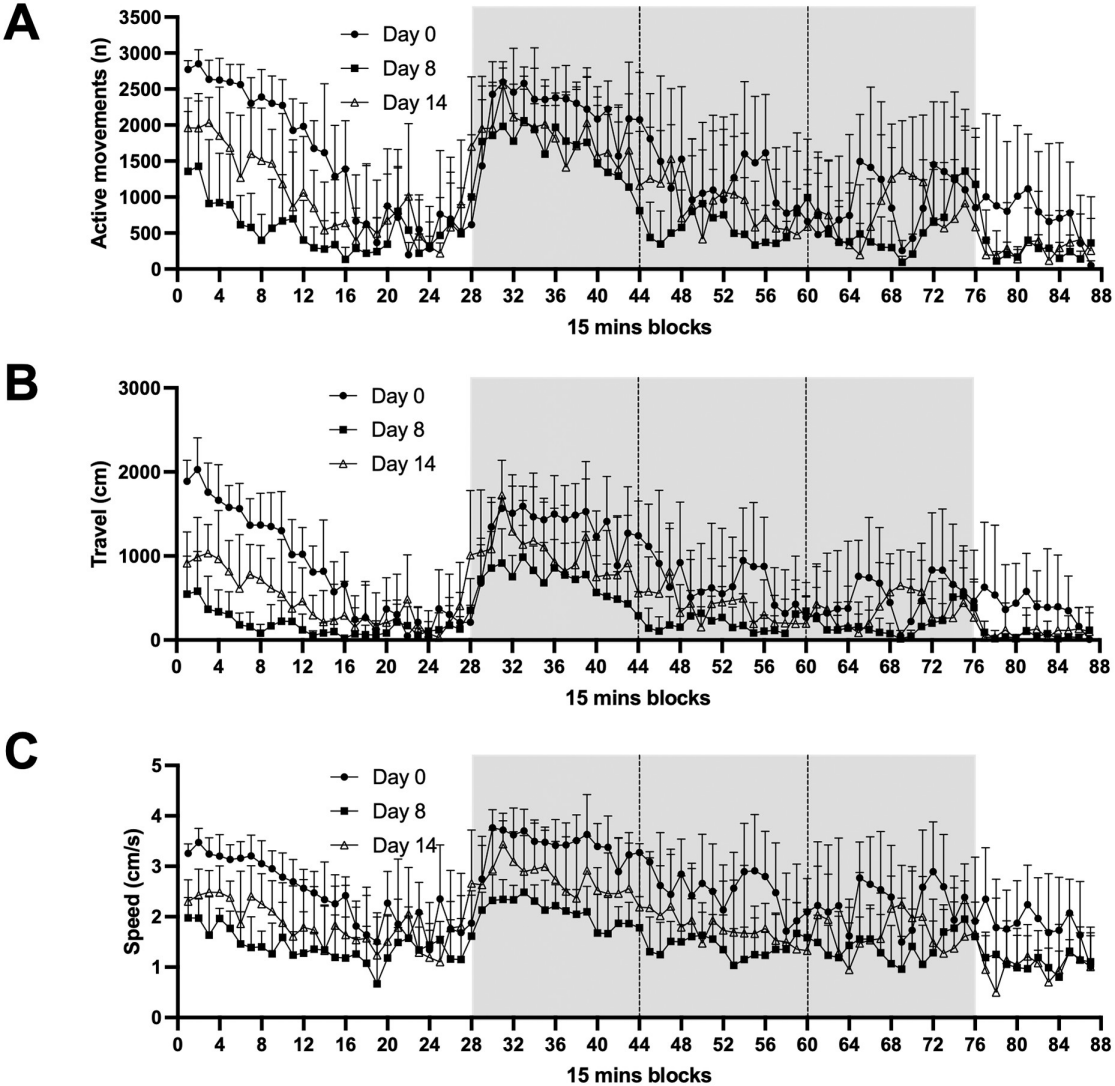
Number of active movements, total distance travelled, and speed of mice with arthritis. Mice were monitored with the smart cage system for 23 h on Day 0, and on Days 8 and 14 after inducing arthritis with arthritogenic K/BxN serum. (**A**) The number of active movements, (**B**) the total distance travelled and (**C**) the mean speed of the animals are shown over the 23 h period divided into 15-minute blocks. Values are means +/- SD, n = 6–8 animals per group. The grey areas represent the night hours (lights out at 7pm until lights on at 7am). The night hours were divided in three blocks of 4 hours.

In addition to monitoring ankle thickness and clinical index, locomotor activity was measured with the smart cage monitoring system for 23-hour periods as described in the Methods section. Time plots for the full 23 hours are shown for three parameters: active movements, distance traveled and speed (Fig 2). The smart cages monitor these parameters continuously, however, to visually depict these activity parameters, Fig 2 shows plots of activity for each 15-minute period during the day in animals at Day 0, Day 7–8 and Day 13–14. The grey area highlights nighttime hours from 7pm to 7am when the lights are off when mice are most active [28].

**Fig 3:**
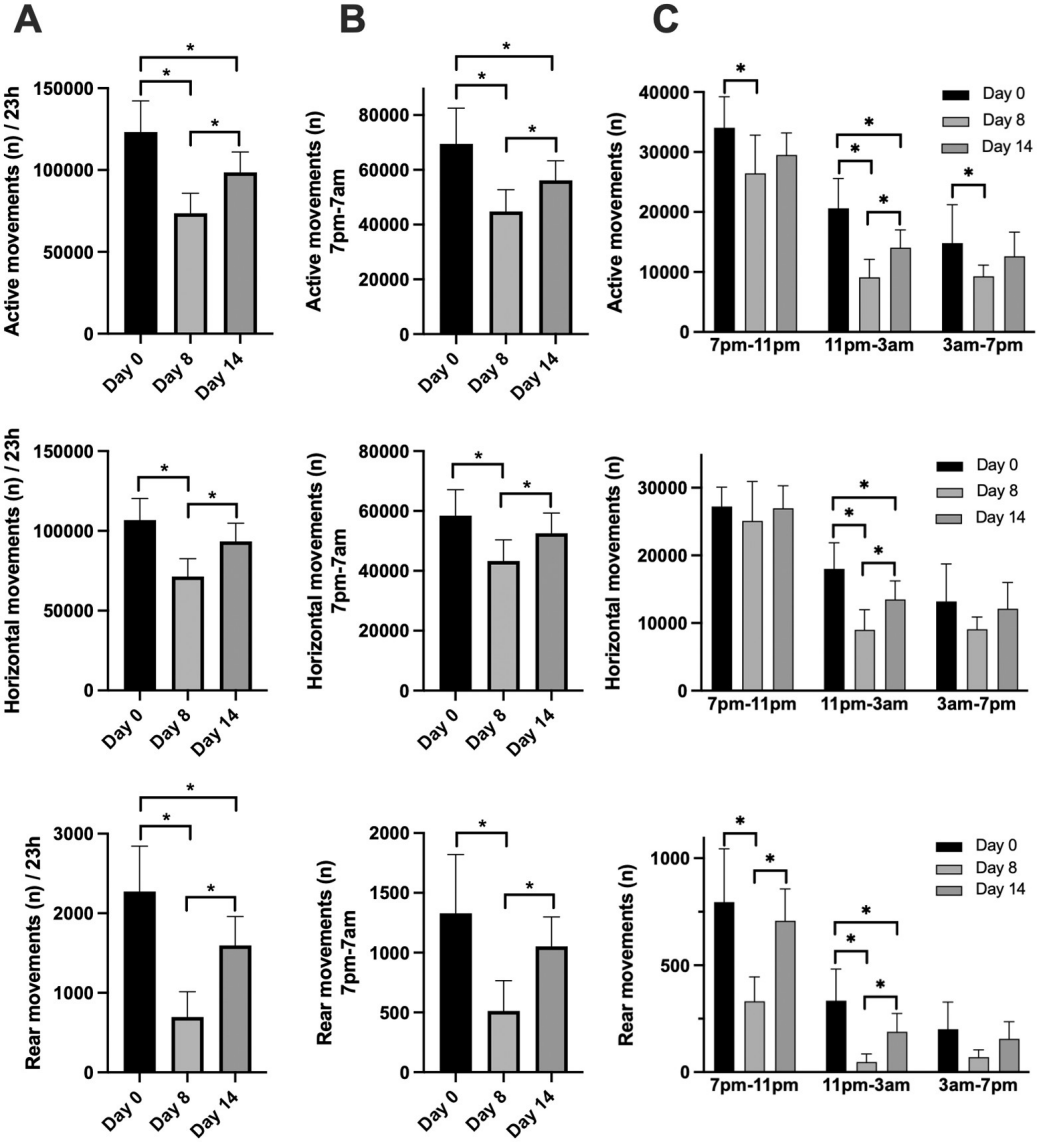
The number of active movements, horizontal movements, and rear movements of mice with arthritis. Mice were monitored with the smart cage system for 23 h on Day 0, and on Days 8 and 14 after inducing arthritis with arthritogenic K/BxN serum. The number of active movements, the number of horizontal movements movements and number of rear movements for the full 23 h period (**A**), from 7pm-7am (lights out—lights on) (**B**), and for the indicated 4 h blocks within the 7pm-7am period (**C**) are shown. Values are means +/- SD, n = 6–8 animals per group. Data in (**A**) and (**B**) were analyzed by 1-way repeated measures analyses. Data in (**C**) were analyzed by 2-way repeated measures analyses. All data were analysed by fitting a mixed effects model since data were missing at day 0 for two of the eight animals. See S2 Table for Mixed effects analysis tables. *Values are different as determined by the Tukey multiple comparisons test, p<0.05.

**Fig 4:**
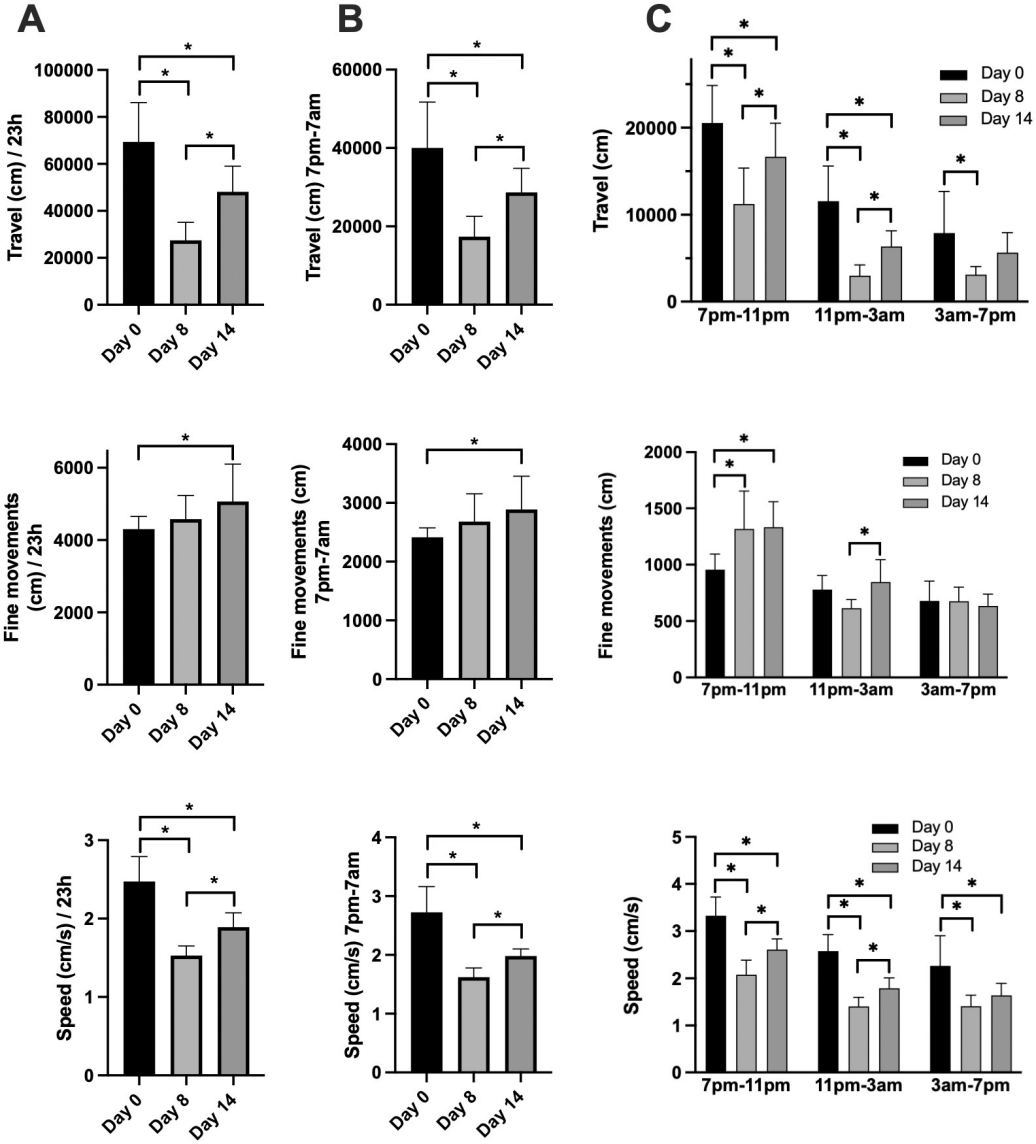
The total distance travelled, the total fine movements distance, and mean speed of mice with arthritis. Mice were monitored with the smart cage system for 23 h on Day 0, and on Days 8 and 14 after inducing arthritis with arthritogenic K/BxN serum. Total distance travelled, total fine movements distance travelled and mean speed for the full 23 h period (**A**), from 7pm-7am (lights out—lights on) (**B**), and for the indicated 4 h blocks within the 7pm-7am period (**C**) are shown. Values are means +/- SD, n = 6–8 animals per group. Data in (**A**) and (**B**) were analyzed by 1-way repeated measures analyses. Data in (**C**) were analyzed by 2-way repeated measures analyses. All data were analysed by fitting a mixed effects model since data were missing at day 0 for two of the eight animals. See S3 Table for Mixed effects analysis tables. *Values are different as determined by the Tukey multiple comparisons test, p<0.05.

To determine whether there were measurable differences in locomotor activity between the phases of illness, several smart cage parameters were compared over the full 23 hours, during the night from 7pm to 7am, and for the three blocks of 4 hours during the night. The number of movements recorded as total active movements, horizontal movements, and rear movements were significantly different in the three phases of the disease (day 0, day 7–8, day 13–14) when measured over the full 23 h period (Fig 3A, S2 Table). Significant differences between disease phases were also observed when measurements were limited to the nighttime hours (7pm–7am) (Fig 3B, S2 Table). Additionally, these locomotor activities were significantly different between the different periods of night, with significant differences also measured between the different phases of disease (Fig 3C, S2 Table). In some instances, the interaction between time of night and phase of disease were also significant. Similar results were also obtained for total distance travelled and speed (cm/s) (Fig 4, S3 Table). For fine movements distance (cm), the only significant difference observed over 24 hours or during the nighttime hours was between Day 0 and Day 14 (Fig 4, S3 Table). Significant differences in fine movements distance were also measured between non-arthritic (Day 0) and arthritic mice during the initial 4-hour nighttime period. Unlike other locomotion parameters, this distance travelled in fine movements was increased in arthritic mice compared to non-arthritic mice on Day 0.

Numerous other parameters related to the amount of time spent on different activities were also measured and are presented in S1–S7 Figs and S4 Table. Generally, similar results were obtained where activity was diminished at Day 8 compared to Day 0, with a partial increase at Day 14. Of note are the more striking changes in rearing behavior in arthritic mice compared to values at Day 0.

### Analysis of a treatment for arthritis with smart cages

After analyzing the progression of arthritis using the smart cage system, the use of this system for the analysis of an intervention known to impact on disease severity was of interest. Since omega-3 polyunsaturated fatty acids (PUFA) from fish oils are known to have anti-inflammatory effects, a diet supplemented with fish oil was designed and fed to arthritic mice. No difference between groups in the weight of the mice was observed. On day 14 the weight of the chow group and fish oil group were 23.8 ± 1.6 g and 24.5 ± 1.6 g (mean ± SD), respectively (p = 0.36, 2-tailed t-test).

**Fig 5:**
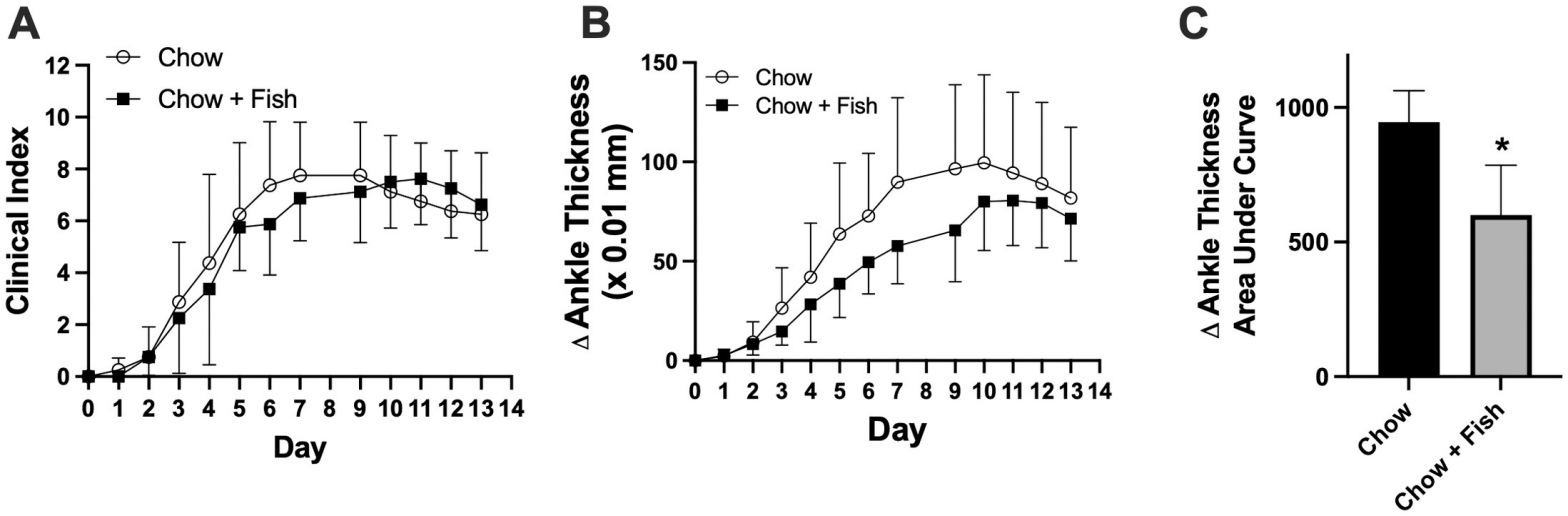
Clinical index and ankle thickness over time of mice consuming chow diets and chow diets supplemented with fish oil. After 3 weeks of experimental diets, arthritis was induced with injections of arthritogenic K/BxN serum into C57BL/6 mice on days 0 and 2. (**A**) Clinical index and (**B**) change in ankle thickness of mouse joints were then measured over time as described in the Methods. Clinical index data were analyzed by 2-way repeated measures ANOVA. Change in ankle thickness data were analyzed by 2-way repeated measures analyses by fitting a mixed effects model. (**C**) Area Under the Curve of changes in ankle thickness over 14 days. *Different from Chow diet determined by unpaired two-tailed t-test, p<0.05. All values are means +/- SD, n = 7–8 animals/group.

Ankle thickness and clinical index were monitored throughout the 14 days of arthritis in mice from both dietary groups (Fig 5). Following a 2-way repeated measures ANOVA analysis, no difference in clinical index between the dietary groups was observed (F (1, 14) = 0.088; p = 0.77) although there was a time effect (F (12, 153) = 59.34; p<0.0001) (Fig 5A). There were also no significant differences measured between diets for ankle thickness determined by 2-way repeated measures analyses by fitting a mixed effects model (F (1, 13) = 2.141; p = 0.167), while the effect of time was significant (F (12, 153) = 59.34; p<0.0001) (Fig 5B). When the overall difference in ankle thickness was quantified with the area under the curve calculations, a significant effect of diet was measured with animals consuming the fish oil diet showing smaller changes in ankle thickness than the chow group (Fig 5C).

**Fig 6:**
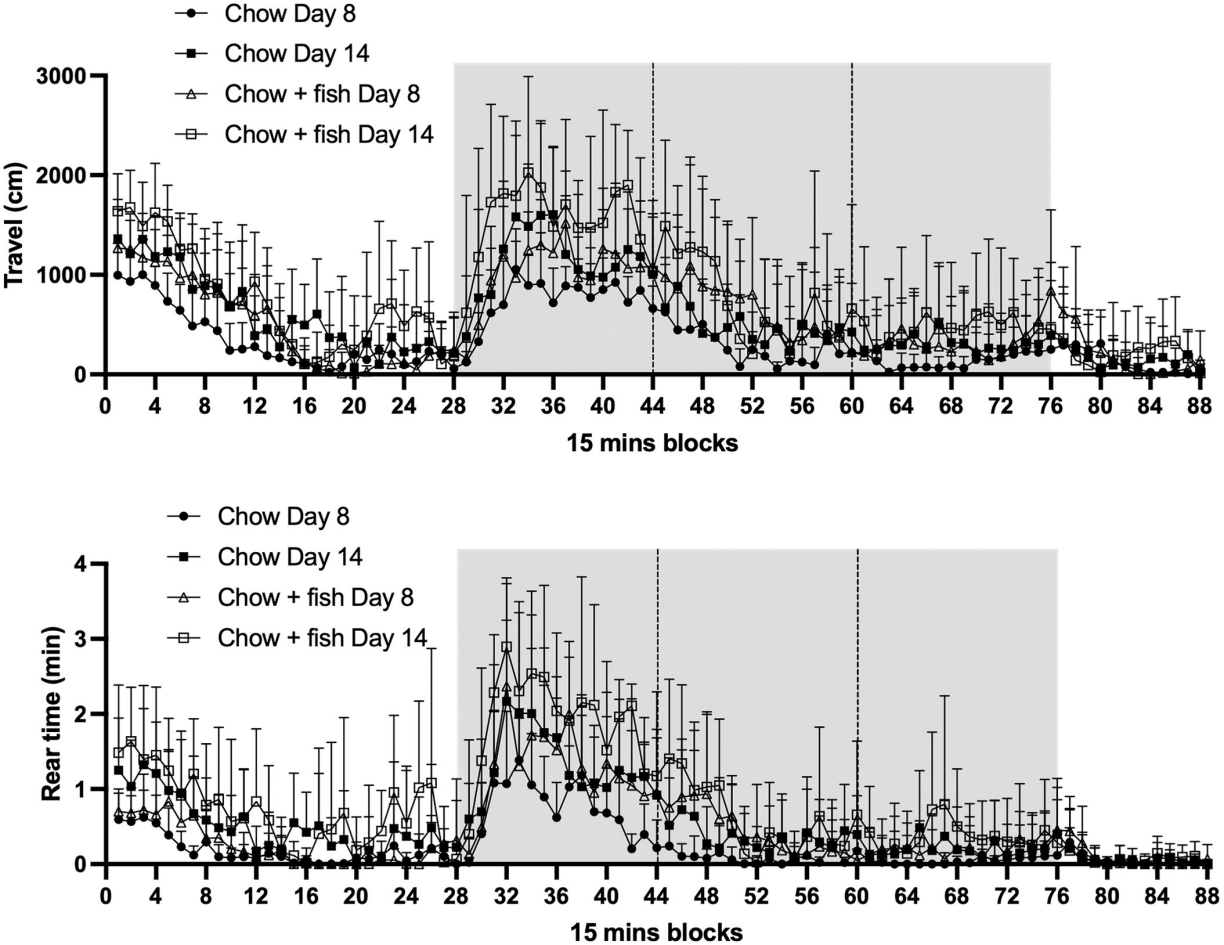
The total distance travelled, and the rear time of mice consuming chow diets and chow diets supplemented with fish oil. Mice consuming chow diets or chow diets supplemented with fish oil were monitored with the smart cage system for 23 h on days 8 and 14 after inducing arthritis with arthritogenic K/BxN serum. Total distance travelled and rear time are shown over the 23h period divided into 15-minute blocks. Values are means +/- SD, n = 8 animals per group. The grey areas represent the night hours (lights out at 7pm until lights on at 7am). The night hours were divided in three blocks of 4 hours for further analysis.

**Fig 7:**
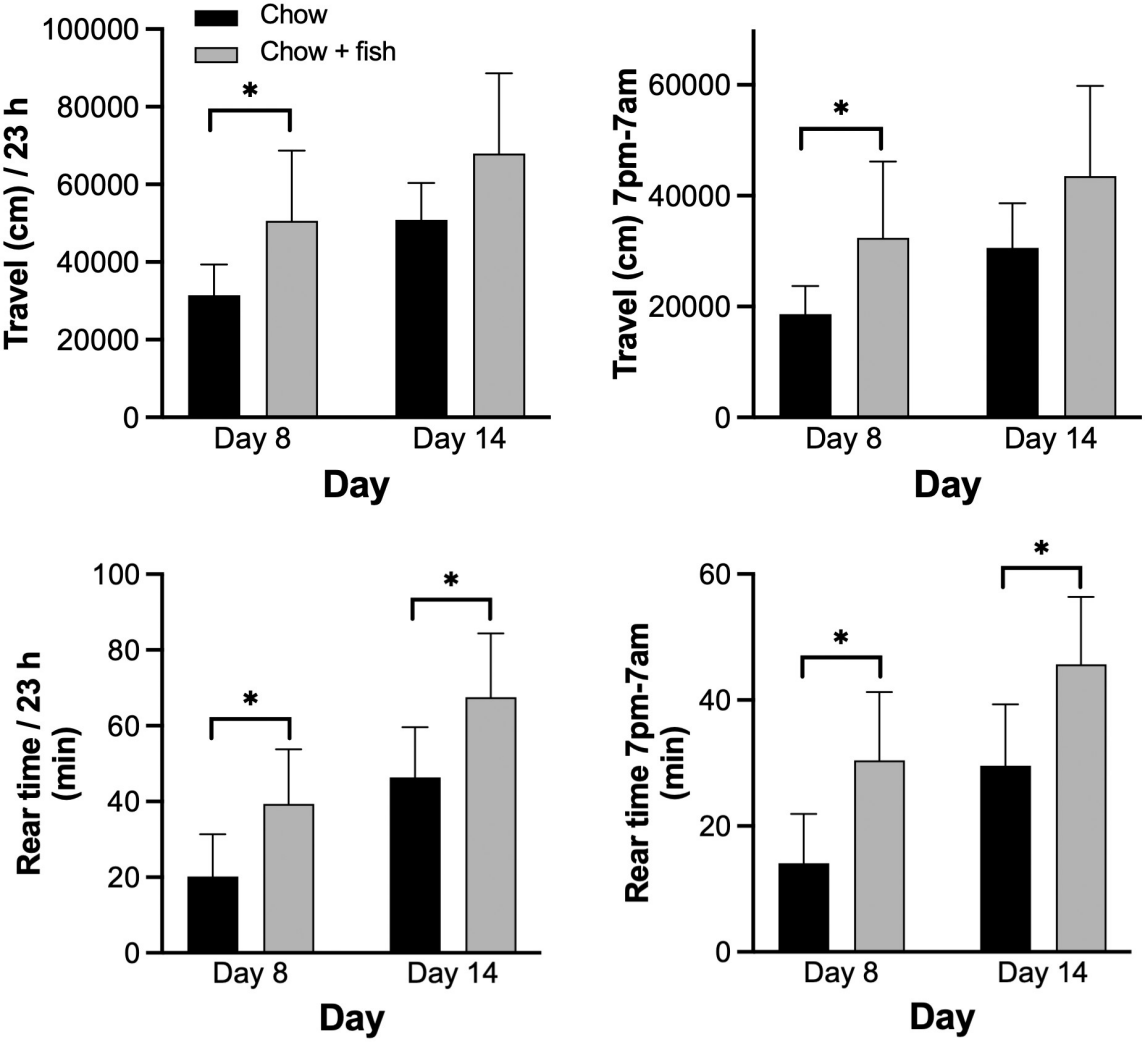
The total distance travelled and the rear time of mice consuming chow diets and chow diets supplemented with fish oil. Mice consuming a chow diet, or a chow diet supplemented with fish oil were monitored with the smart cage system for 23 h on days 8 and 14 after inducing arthritis with arthritogenic K/BxN serum. Total distance travelled and the rear time for the full 23h period, and from 7pm-7am (lights out—lights on) are shown. Values are means +/- SD, n = 8 animals per group. * Values are different determined by two-way repeated measures ANOVA tests with Šídák’s multiple comparison analyses, p<0.05. See S5 Table for ANOVA tables.

In addition to measuring clinical index and ankle thickness, mice were also monitored in smart cages for 23-hour periods on Days 8 and 14. Plots for distance travelled and rear time are demonstrated for each Day for the two diets (Fig 6). The same trend as shown in Fig 2 is observed: a high activity in the first hours and another maximum of activity at the beginning of the night (Fig 6). Again, to dissect the analysis of different locomotor parameters at the two different days of arthritis, the total distance travelled and the time spent rearing (rear time) were compared between the two dietary groups (Fig 7, S5 Table). The group consuming the chow diet travelled a significantly smaller distance over the 23 h period and during the night period at Day 8 compared to the fish oil group. Additionally, the fish oil group spent significantly more time rearing than the chow group at both Days 8 and 14. Results of other measured parameters are shown in S8 and S9 Figs and S6 Table where several significant differences between dietary groups were measured. Of note, dietary treatment had no significant effect on fine movements. Lastly, there were no significant dietary group x disease phase interactions (S5 and S6 Tables).

## Discussion

The current study describes the ability to monitor locomotor activity of arthritic mice over prolonged periods in cages where they have their regular access to food, water, and bedding material. Thus, this measurement of their everyday activities can be an indication of their well-being and disease burden. The use of the smart cage system, which was developed and has been used to measure neuropsychological behavior or the impact of neurological injury on mobility [19–24], had not previously been used to assess locomotor activity of mice with inflammatory arthritis. Overall, many aspects of their movements and behavior were significantly affected by the induction of inflammatory arthritis in a time-dependent manner. Importantly, the use of this smart cage system was shown to be a potentially valuable tool for the evaluation of the efficacy of therapeutic interventions.

Arthritis was induced in mice by injections of K/BxN serum and the mice were then monitored for 14 consecutive days by measuring the clinical index score and the ankle thickness as is typically done to evaluate disease progression in this model [25, 26, 29]. As expected, a gradual increase in inflammation was measured that plateaued from Day 9 to Day 11, with what appeared to be a beginning of resolution measured on Day 13 (Fig 1). To analyze the different stages of the disease based on locomotor activity with the smart cage system, mice were monitored for a period of 23 hours from Days -1 to 0, 7 to 8 and 13 to 14 of the disease. Various locomotor activity plots with smart cages over 23-hour periods showed that the locomotor activities of can be continuously monitored (Fig 2 and S1–S3 Figs). These plots show that mice measured on Day 0 appeared to have the highest activity level with the lowest apparent activity levels measured on Day 8, while the activity levels of Day 14 were intermediate.

To better evaluate smart cage results, the sum or average of each locomotor activity were calculated for the full 23-hour period, during the 12-hour period of darkness and during each 4-hour tranche of the period of darkness. This was done for two reasons. Firstly, when the animals were transferred to smart cages, their activity levels were elevated for approximately 3–4 hours until they settled down in their new cage. Secondly, it was reasoned that isolating the periods of darkness may be a valuable measure since this is when these primarily nocturnal animals are most active [28], especially during the first few hours of darkness. Several observations could be made regarding the impact of arthritis on locomotor activity. The number of activity movements, or how often sensors are triggered, were significantly decreased on Day 8 compared to Day 0 over the full 23-hour period and during the 12-hour dark period, with a significant increase in movements on Day 14 suggesting that the animals are experiencing less pain during what could be considered the resolution phase of inflammation (Fig 3). It is noteworthy that during the first 4 hours of darkness when animals are most active, horizontal movements, which are the most abundant movements, are similar on all three monitoring days. However, during the next 4-hour period (11pm–3am), while animals are nevertheless still quite active on Day 0, a significant decrease in activity was measured on Day 8 and Day 14, although horizontal movements on Day 14 were significantly greater than on Day 8. This suggests that while arthritic pain did not affect the animals’ horizontal exploration when lights went out, the arthritic animals could not sustain the activity level for a prolonged period compared to non-arthritic animals. Moreover, the actual distance travelled by the animals (Fig 4) was affected by arthritis during all periods of the day and was seemingly more pronounced than the impact on the number of horizontal activity movements. This apparent discrepancy is because distance travelled is a function of both the number of activity movements and the speed of the animals’ movements which was also impacted by arthritis.

Rearing activity movements on the other hand, which is when animals rear up onto their hind paws presumably to eat, was severely impacted by arthritis during the 23-hour monitoring periods, including the 4-hour tranches of time during darkness. In fact, rearing appeared to be the activity that was most impacted by arthritis at Day 8 whether measured as the number of rear movements (Fig 3) or the amount of time spent rearing (S5 Fig). Rear movements were significantly restored on Day 14 compared to Day 8, to the extent that total rear movements during the 12-hour lights-out period were not different from those on Day 0. However, the time spent rearing was still significantly decreased on Day 14 compared to Day 0 (S5 Fig) indicating that although the number of rearing events were restored, the pain exhibited during rearing likely did not allow the animal to maintain this position.

Unlike other locomotor activities, distance associated with fine movements as well as the time spent performing fine movements were either unchanged after the induction of arthritis, or even increased compared to Day 0 as measured during the first 4 hours of darkness (Fig 4 and S5 Fig). This could be explained by the fact that when the mice are subjected to more arthritic pain, they may tend to become more sedentary increasing the frequency of smaller movements such as grooming than when they are healthy. Finally, no differences were observed in the number of net rotations (S4 and S7 Figs) indicating that the induction of arthritis did not influence this parameter that is often altered following brain injury [30].

Overall, these results demonstrate that changes in several parameters of locomotor activities are associated with the development of arthritis, that these changes may be more pronounced at different times of the day and that significant improvements in locomotor activity associated with the resolution of an arthritic episode can be ascertained. Indeed, aspects of the progression of arthritis, such as resolution, were clearly shown using several parameters with smart cages, whereas the clinical index and ankle thickness measurements only suggested, at best, that resolution had begun.

The ability to monitor the various locomotor parameters of arthritic mice are potentially of use in pre-clinical studies. Unlike ankle thickness and clinical index that essentially measure the physical appearance of the paws and ankle joints, a real time measure of the movements of these arthritic animals provides data showing biologically relevant changes in behavior. Indeed, a 60% decrease in the distance travelled over the course of the day, or an 85% decrease in the amount of time spent rearing are behavioral changes that are clinically relevant in arthritis.

Having established the changes in locomotor activity that were associated with the development of arthritis, it was of interest to test, as a proof of concept, whether locomotor activity changes resulting from a therapeutic intervention could be measured. Rather than evaluating a pharmaceutical intervention such as non-steroidal anti-inflammatory drugs that can potentially impact on behavior due to an analgesic effect, a preventative dietary intervention was utilized that has been previously shown to impact on disease progression in the K/BxN serum transfer model of inflammatory arthritis [31], and which has been shown to impact on the severity of RA in human trials [32, 33]. Therefore, locomotor activity was measured in mice that had consumed a regular chow diet and in mice having consumed a diet supplemented with fish oil rich in omega-3 fatty acids which can impact on cytokine and lipid mediator production, and are the precursors of specialized pro-resolving mediators of inflammation [34, 35].

While the fish oil diet had no measurable impact on clinical index, increases in ankle thickness were significantly attenuated compared to the chow-fed group (Fig 5) in area-under curve analyses, although no significant effect of diet was observed in repeated measures analyses of changes in ankle thickness over time with no apparent difference between diets during the resolution phase. This was somewhat surprising since omega-3 PUFA are precursors of the specialized pro-resolving mediators of inflammation. The two locomotor activities that were most affected by arthritis, travel distance and rear time as discussed above, were also impacted by diets with significant increases in the fish oil group compared to the chow-fed group during the peak of arthritis at Day 8 (Fig 7). In fact, the distance traveled and time spent rearing were approximately 60% and 100% greater, respectively, in the fish oil group than the chow group during the peak of disease at Day 8. Such increases in activity would be deemed clinically significant in humans, indicating that the smart cage system is of value in pre-clinical studies. On Day 14, the fish oil diet group showed a tendency for greater distances travelled, whereas rearing time was significantly increased compared to the chow-fed group. Other, but not all, locomotor activity parameters also showed improvement or tendency for improvements in the fish oil group, at both Day 8 and Day 14.

Overall, locomotor activity measured with the smart cage system was able to measure diet-induced changes in disease burden that were not captured using the clinical index or ankle thickness measurements. Indeed, despite significant changes in locomotor activity associated with dietary modification, the clinical index failed to measure any changes in disease burden. This highlights the limitations of relying on the clinical index as a measure of arthritis. One disadvantage with this measure is the great subjectivity associated with clinical index scores. Additionally, two different mice may have the same clinical index score, but one may have significantly more swollen paws than the other since the index attributes a score based number of sites on the paw showing inflammation (phalanx joints, wrist/ankle, or on the surface of the paw), but not the extent of inflammation at each site [10, 36]. Similarly, the ankle thickness failed to detect differences during the resolution phase of the disease whereas several locomotor activity parameters showed significant differences.

To conclude, the current study demonstrated that the smart cage system has the potential to be an important tool in the study of inflammatory arthritis in rodent models. This system can measure biologically-relevant changes in arthritis over time and in the context of treatment suggesting clinical relevance in pre-clinical studies. Furthermore, this system identified periods of day where disease burden was most apparent, as well as the animals’ locomotor activities that were most affected by arthritis and by a preventative dietary intervention. Although the current study was limited to one mouse model of inflammatory arthritis, the results suggest that this system would likely be of use in different animal models and such evaluations are warranted. In short, much more information regarding disease progression and the efficacy of therapeutic interventions can be obtained using this smart cage system than with other methods commonly used to measure arthritis in animal models.

## Supporting information

S1 Table Fatty acid profiles of experimental diets. (PDF)

S2 Table Mixed effects analysis tables for the indicated parameters. Comparisons of disease phases (day 0, day 8, day 14) over the full 23 hours, or over the 7pm-7am period were analyzed by 1-way repeated measures analyses. Comparisons of disease phases (day 0, day 8, day 14) over the different times of the night were performed by 2-way repeated measures analyses. All data were analysed by fitting a mixed effects model since data were missing at day 0 for two of the eight animals.
(PDF)

S3 Table Mixed effects analysis tables for the indicated parameters. Comparisons of disease phases (day 0, day 8, day 14) over the full 23 hours, or over the 7pm-7am period were analyzed by 1-way repeated measures analyses. Comparisons of disease phases (day 0, day 8, day 14) over the different times of the night were performed by 2-way repeated measures analyses. All data were analysed by fitting a mixed effects model since data were missing at day 0 for two of the eight animals.
(PDF)

S4 Table Mixed effects analysis tables for the indicated parameters. Comparisons of disease phases (day 0, day 8, day 14) over the full 23 hours, or over the 7pm-7am period were analyzed by 1-way repeated measures analyses. Comparisons of disease phases (day 0, day 8, day 14) over the different times of the night were performed by 2-way repeated measures analyses. All data were analysed by fitting a mixed effects model since data were missing at day 0 for two of the eight animals.
(PDF)

S5 Table ANOVA tables for the indicated parameters. Comparisons of disease phases (day 8, day 14) for the two dietary groups were performed by repeated measures 2-way ANOVA.
(PDF)

S6 Table ANOVA tables for the indicated parameters. Comparisons of disease phases (day 8, day 14) for the two dietary groups were performed by repeated measures 2-way ANOVA.
(PDF)

S1 Fig Number of horizontal movements, total fine movements, and the travel time of mice with arthritis. Mice were monitored with the smart cage system for 23 h on Day 0, and on Days 8 and 14 after inducing arthritis with arthritogenic K/BxN serum. (**A**) The number of horizontal movements, (**B**) the number of fine movements and (**C**) the travel time of the animals are shown over the 23h period divided into 15-minute blocks. Values are means +/- SD, n = 6–8 animals per group. The grey areas represent the night hours (lights out at 7pm until lights on at 7am). The night hours were divided in three blocks of 4 hours.
(TIFF)

S2 Fig Total active time, rear time, horizontal movements time, and fine movements time of mice with arthritis. Mice were monitored with the smart cage system for 23 h on Day 0, and on Days 8 and 14 after inducing arthritis with arthritogenic K/BxN serum. (**A**) The active time, (**B**) the rearing time, (**C**) the horizontal movements time, and (**D**) the fine movements time of the animals are shown over the 23h period divided into 15-minute blocks. Values are means +/- SD, n = 6–8 animals per group. The grey areas represent the night hours (lights out at 7pm until lights on at 7am). The night hours were divided in three blocks of 4 hours.
(TIFF)

S3 Fig The percentage of active time, rear time, and horizontal movements time of mice with arthritis. Mice were monitored with the smart cage system for 23 h on Day 0, and on Days 8 and 14 after inducing arthritis with arthritogenic K/BxN serum. (**A**) The percent of active time, (**B**) the percent of rearing time, and (**C**) the percent of horizontal movements time are shown over the 23h period divided into 15-minute blocks. Values are means +/- SD, n = 6–8 animals per group. The grey areas represent the night hours (lights out at 7pm until lights on at 7am). The night hours were divided in three blocks of 4 hours.
(TIFF)

S4 Fig Number of lefts, rights, and net rotations of mice with arthritis. Mice were monitored with the smart cage system for 23 h on Day 0, and on Days 8 and 14 after inducing arthritis with arthritogenic K/BxN serum. (**A**) The number of left rotations, (**B**) the number of right rotations, and (**C**) the number of net rotations are shown over the 23h period divided into 15-minute blocks. Values are means +/- SD, n = 6–8 animals per group. The grey areas represent the night hours (lights out at 7pm until lights on at 7am). The night hours were divided in three blocks of 4 hours.
(TIFF)

S5 Fig The total active time, rear time, horizontal movements time, and fine movements time of mice with arthritis. Values for the full 23 h period (**A**), from 7pm-7am (lights out—lights on) (**B**), and for the indicated 4 h blocks within the 7pm-7am period (**C**) are shown. Values are means +/- SD, n = 6–8 animals per group. Data in (**A**) and (**B**) were analyzed by 1-way repeated measures analyses. Data in (**C**) were analyzed by 2-way repeated measures analyses. All data were analysed by fitting a mixed effects model since data were missing at day 0 for two of the eight animals. See S4 Table for Mixed effects analysis tables. *Values are different as determined by the Tukey multiple comparisons test, p<0.05.
(TIFF)

S6 Fig The percentage of active time, rear time, and horizontal movements time of mice with arthritis. Values for the full 23 h period (**A**), from 7pm-7am (lights out—lights on) (**B**), and for the indicated 4 h blocks within the 7pm-7am period (**C**) are shown. Values are means +/- SD, n = 6–8 animals per group. Data in (**A**) and (**B**) were analyzed by 1-way repeated measures analyses. Data in (**C**) were analyzed by 2-way repeated measures analyses. All data were analysed by fitting a mixed effects model since data were missing at day 0 for two of the eight animals. See S4 Table for Mixed effects analysis tables. *Values are different as determined by the Tukey multiple comparisons test, p<0.05.
(TIFF)

S7 Fig The total lefts, rights, and net rotations of mice with arthritis. Values for the full 23 h period (**A**), from 7pm-7am (lights out—lights on) (**B**), and for the indicated 4 h blocks within the 7pm-7am period (**C**) are shown. Values are means +/- SD, n = 6–8 animals per group. Data in (**A**) and (**B**) were analyzed by 1-way repeated measures analyses. Data in (**C**) were analyzed by 2-way repeated measures analyses. All data were analysed by fitting a mixed effects model since data were missing at day 0 for two of the eight animals. See S4 Table for Mixed effects analysis tables. *Values are different as determined by the Tukey multiple comparisons test, p<0.05.
(TIFF)

S8 Fig Impact of a diet supplemented with fish oil on the number of active, horizontal and rear movements, and fine movements distance of mice with arthritis. Mice consuming a chow diet, or a chow diet supplemented with fish oil were monitored with the smart cage system for 23 h on days 8 and 14 after inducing arthritis with arthritogenic K/BxN serum. Total distance travelled and the number of active movements for the full 23h period, and from 7pm-7am (lights out—lights on) are shown. Values are means +/- SD, n = 8 animals per group. * Values are different determined by two-way repeated measures ANOVA tests with Šídák’s multiple comparison analyses, p<0.05. See S6 Table for ANOVA tables.
(TIFF)

S9 Fig Impact of a diet supplemented with fish oil on active time, horizontal movements time, fine movements time and speed of mice with arthritis. Mice consuming a chow diet, or a chow diet supplemented with fish oil were monitored with the smart cage system for 23 h on days 8 and 14 after inducing arthritis with arthritogenic K/BxN serum. Total time spend on each activity and mean speed for the full 23h period, and from 7pm-7am (lights out—lights on) are shown. Values are means +/- SD, n = 8 animals per group. * Values are different determined by two-way repeated measures ANOVA tests with Šídák’s multiple comparison analyses, p<0.05. See S6 Table for ANOVA tables.
(TIFF)

S1 File Smart cage data. (XLSX)

S2 File Smart cage data chow diets days 7–8. (XLSX)

S3 File Smart cage data chow diets days 13–14. (XLSX)

S4 File Smart cage data chow+fish diets days 7–8. (XLSX)

S5 File Smart cage data chow+fish diets days 13–14. (XLSX)
