# Supplementary material for: Locomotor activity as an effective measure of the severity of inflammatory arthritis in a mouse model
Source: PLoS One. 2024 Jan 17;19(1):e0291399. doi: 10.1371/journal.pone.0291399 (PMC10793911; doi:10.1371/journal.pone.0291399)
Supplement: S1 Table — (PDF) [file pone.0291399.s001.pdf]

S1 Table. Fatty acid profiles of experimental diets determined by gas chromatography with FID detection.

Fatty Acids	Diets	
	Chow	Chow + fish
**16:0**	17.7 ± 0.1	20.1 ± 0.1
**18:0**	7.8 ± 0.1	4.4 ± 0.0
**18:1 n-9**	28.4 ± 0.2	11.7 ± 0.1
**18:2 n-6**	37.0 ± 0.1	9.8 ± 0.0
**18:3 n-3**	4.5 ± 0.0	2.5 ± 0.0
**18:4 n-3**	0.0 ± 0.0	2.6 ± 0.0
**20:4 n-6**	0.1 ± 0.0	0.9 ± 0.1
**20:5 n-3**	0.0 ± 0.0	11.3 ± 0.1
**22:5 n-3**	0.0 ± 0.0	1.8 ± 0.0
**22:6 n-3**	0.0 ± 0.0	7.3 ± 0.1

Values are mean % of total FAME (mol %) ± SD, n=4 samples analysed per diet.
