# Supplementary material for: Locomotor activity as an effective measure of the severity of inflammatory arthritis in a mouse model
Source: PLoS One. 2024 Jan 17;19(1):e0291399. doi: 10.1371/journal.pone.0291399 (PMC10793911; doi:10.1371/journal.pone.0291399)
Supplement: S2 Table — Comparisons of disease phases (day 0, day 8, day 14) over the full 23 hours, or over the 7pm-7am period were analyzed by 1-way repeated measures analyses. Comparisons of disease phases (day 0, day 8, day 14) over the different times of the night were performed by 2-way repeated measures analyses. All data were analysed by fitting a mixed effects model since data were missing at day 0 for two of the eight animals. (PDF) [file pone.0291399.s002.pdf]

S2 Table. Mixed effects analysis tables for the indicated parameters. Comparisons of disease phases (day 0, day 8, day 14) over the full 23 hours, or over the 7pm-7am period were analyzed by 1-way repeated measures analyses. Comparisons of disease phases (day 0, day 8, day 14) over the different times of the night were performed by 2-way repeated measures analyses. All data were analysed by fitting a mixed effects model since data were missing at day 0 for two of the eight animals.

**Parameter**	**F (DFn, DFd)**	**P value**
Active movements (n) / 23 h	F (2, 12) = 32.37	<0.0001
Active movements (n) 7pm-7am	F (2, 12) = 21.10	0.0001
Time of night	F (2, 14) = 138.5	<0.0001
Day	F (2, 14) = 21.10	<0.0001
Time of night x Day	F (4, 22) = 1.143	0.3625
**Parameter**	**F (DFn, DFd)**	**P value**
Horiz. movements (n) / 23 h	F (2, 12) = 23.11	<0.0001
Horiz. movements (n) 7pm-7am	F (2, 12) = 13.60	0.0008
Time of night	F (2, 14) = 117.5	<0.0001
Day	F (2, 14) = 12.00	0.0009
Time of night x Day	F (4, 22) = 1.810	0.1628
**Parameter**	**F (DFn, DFd)**	**P value**
Rear movements (n) / 23 h	F (2, 12) = 50.79	<0.0001
Rear movements 7pm-7am	F (2, 12) = 16.30	0.0004
Time of night	F (2, 14) = 204.5	<0.0001
Day	F (2, 14) = 19.46	<0.0001
Time of night x Day	F (4, 21) = 9.094	0.0002
