# Supplementary material for: Locomotor activity as an effective measure of the severity of inflammatory arthritis in a mouse model
Source: PLoS One. 2024 Jan 17;19(1):e0291399. doi: 10.1371/journal.pone.0291399 (PMC10793911; doi:10.1371/journal.pone.0291399)
Supplement: S3 Table — Comparisons of disease phases (day 0, day 8, day 14) over the full 23 hours, or over the 7pm-7am period were analyzed by 1-way repeated measures analyses. Comparisons of disease phases (day 0, day 8, day 14) over the different times of the night were performed by 2-way repeated measures analyses. All data were analysed by fitting a mixed effects model since data were missing at day 0 for two of the eight animals. (PDF) [file pone.0291399.s003.pdf]

S3 Table. Mixed effects analysis tables for the indicated parameters. Comparisons of disease phases (day 0, day 8, day 14) over the full 23 hours, or over the 7pm-7am period were analyzed by 1-way repeated measures analyses. Comparisons of disease phases (day 0, day 8, day 14) over the different times of the night were performed by 2-way repeated measures analyses. All data were analysed by fitting a mixed effects model since data were missing at day 0 for two of the eight animals.

**Parameter**	**F (DFn, DFd)**	**P value**
Travel (cm) / 23 h	F (2, 12) = 43.88	<0.0001
Travel (cm) 7pm-7am	F (2, 12) = 32.80	0.0001
Time of night	F (2, 14) = 131.4	<0.0001
Day	F (2, 14) = 32.80	<0.0001
Time of night x Day	F (4, 22) = 2.219	0.1000
**Parameter**	**F (DFn, DFd)**	**P value**
Fine move. (cm) / 23 h	F (2, 12) = 4.589	0.0331
Fine move. (cm) 7pm-7am	F (2, 12) = 6.300	0.0135
Time of night	F (2, 14) = 62.82	<0.0001
Day	F (2, 14) = 3.988	0.0426
Time of night x Day	F (4, 20) = 5.208	0.0049
**Parameter**	**F (DFn, DFd)**	**P value**
Speed / 23 h	F (2, 12) = 51.95	<0.0001
Speed 7pm-7am	F (2, 12) = 44.65	<0.0001
Time of night	F (2, 14) = 94.41	<0.0001
Day	F (2, 14) = 46.42	<0.0001
Time of night x Day	F (4, 21) = 1.842	0.1585
