# Supplementary material for: Locomotor activity as an effective measure of the severity of inflammatory arthritis in a mouse model
Source: PLoS One. 2024 Jan 17;19(1):e0291399. doi: 10.1371/journal.pone.0291399 (PMC10793911; doi:10.1371/journal.pone.0291399)
Supplement: S4 Table — Comparisons of disease phases (day 0, day 8, day 14) over the full 23 hours, or over the 7pm-7am period were analyzed by 1-way repeated measures analyses. Comparisons of disease phases (day 0, day 8, day 14) over the different times of the night were performed by 2-way repeated measures analyses. All data were analysed by fitting a mixed effects model since data were missing at day 0 for two of the eight animals. (PDF) [file pone.0291399.s004.pdf]

S4 Table. Mixed effects analysis tables for the indicated parameters. Comparisons of disease phases (day 0, day 8, day 14) over the full 23 hours, or over the 7pm-7am period were analyzed by 1-way repeated measures analyses. Comparisons of disease phases (day 0, day 8, day 14) over the different times of the night were performed by 2-way repeated measures analyses. All data were analysed by fitting a mixed effects model since data were missing at day 0 for two of the eight animals.

**Parameter**	**F (DFn, DFd)**	**P value**
Active time (min) / 23 h	F (2, 12) = 32.37	P<0.0001
Active time (min) 7pm-7am	F (2, 12) = 21.10	P=0.0001
Time of night	F (2, 14) = 138.6	<0.0001
Day	F (2, 14) = 21.10	<0.0001
Time of night x Day	F (4, 22) = 1.143	0.3625
**Parameter**	**F (DFn, DFd)**	**P value**
Rear time (min) / 23 h	F (2, 12) = 49.79	P<0.0001
Rear time (min) 7pm-7am	F (2, 12) = 28.16	P<0.0001
Time of night	F (2, 14) = 69.22	<0.0001
Day	F (2, 14) = 28.16	<0.0001
Time of night x Day	F (4, 22) = 13.91	<0.0001
**Parameter**	**F (DFn, DFd)**	**P value**
Horiz. movements (min) / 23 h	F (2, 12) = 23.25	P<0.0001
Horiz. movements (min) 7pm-7am	F (2, 12) = 13.56	P=0.0008
Time of night	F (2, 14) = 117.3	<0.0001
Day	F (2, 14) = 11.97	0.0009
Time of night x Day	F (4, 22) = 1.796	0.1656
**Parameter**	**F (DFn, DFd)**	**P value**
Fine movements (min) / 23 h	F (2, 12) = 4.868	P=0.0283
Fine movements (min) 7pm-7am	F (2, 12) = 5.658	P=0.0186
Time of night	F (2, 14) = 40.24	<0.0001
Day	F (2, 14) = 4.279	0.0355
Time of night x Day	F (4, 22) = 3.713	0.0186
**Parameter**	**F (DFn, DFd)**	**P value**
Active time (%) / 23 h	F (2, 12) = 32.36	P<0.0001
Active time (%) 7pm-7am	F (2, 12) = 21.79	P=0.0001
Time of night	F (2, 14) = 138.6	<0.0001
Day	F (2, 14) = 21.10	<0.0001
Time of night x Day	F (4, 22) = 1.143	0.3623
**Parameter**	**F (DFn, DFd)**	**P value**
Rear time (%) / 23 h	F (2, 12) = 49.77	P<0.0001

Rear time (%) 7pm-7am	F (2, 12) = 28.20	P<0.0001
Time of night	F (2, 14) = 69.26	<0.0001
Day	F (2, 14) = 28.14	<0.0001
Time of night x Day	F (4, 22) = 13.93	<0.0001
**Parameter**	**F (DFn, DFd)**	**P value**
Horiz. movement time (%) / 23 h	F (2, 12) = 23.10	P<0.0001
Horiz. movement time (%) 7pm-7am	F (2, 12) = 14.39	P=0.0006
Time of night	F (2, 14) = 117.5	<0.0001
Day	F (2, 14) = 12.00	0.0009
Time of night x Day	F (4, 22) = 1.812	0.1626
**Parameter**	**F (DFn, DFd)**	**P value**
Left rotations (n) / 23 h	F (2, 12) = 13.65	P=0.0008
Left rotations (n) 7pm-7am	F (2, 12) = 4.168	P=0.0316
Time of night	F (2, 14) = 67.70	<0.0001
Day	F (2, 14) = 4.168	0.0380
Time of night x Day	F (4, 22) = 1.303	0.2997
**Parameter**	**F (DFn, DFd)**	**P value**
Right rotations (n) / 23 h	F (2, 11) = 6.652	P=0.0128
Right rotations (n) 7pm-7am	F (2, 12) = 1.680	P=0.2273
Time of night	F (2, 14) = 41.81	<0.0001
Day	F (2, 14) = 1.680	0.2218
Time of night x Day	F (4, 22) = 0.462	0.7626
**Parameter**	**F (DFn, DFd)**	**P value**
Net rotations (n) / 23 h	F (2, 19) = 0.277	P=0.761
Net rotations (n) 7pm-7am	F (2, 12) = 0.753	P=0.492
Time of night	F (2, 14) = 0.165	0.8496
Day	F (2, 14) = 0.753	0.4889
Time of night x Day	F (4, 22) = 0.197	0.9371
