# Supplementary material for: Locomotor activity as an effective measure of the severity of inflammatory arthritis in a mouse model
Source: PLoS One. 2024 Jan 17;19(1):e0291399. doi: 10.1371/journal.pone.0291399 (PMC10793911; doi:10.1371/journal.pone.0291399)
Supplement: S5 Table — Comparisons of disease phases (day 8, day 14) for the two dietary groups were performed by repeated measures 2-way ANOVA. (PDF) [file pone.0291399.s005.pdf]

S5 Table. ANOVA tables for the indicated parameters. Comparisons of disease phases (day 8, day 14) for the two dietary groups were performed by repeated measures 2-way ANOVA.

**Travel (cm) / 23 h**	**F (DFn, DFd)**	**P value**
Day x Diet	F (1, 14) = 0.09393	P=0.7638
Day	F (1, 14) = 29.46	P<0.0001
Diet	F (1, 14) = 7.221	P=0.0177
**Travel (cm) / 7pm-7am**		
Day x Diet	F (1, 14) = 0.02202	P=0.8842
Day	F (1, 14) = 18.38	P=0.0008
Diet	F (1, 14) = 6.639	P=0.0220

**Rear movements (min) / 23 h**	**F (DFn, DFd)**	**P value**
Day x Diet	F (1, 14) = 0.08900	P=0.7698
Day	F (1, 14) = 62.86	P<0.0001
Diet	F (1, 14) = 10.73	P=0.0055
**Rear movements (min) / 7pm-7am**		
Day x Diet	F (1, 14) = 0.0024	P=0.9615
Day	F (1, 14) = 33.08	P<0.0001
Diet	F (1, 14) = 15.36	P=0.0015
