# Supplementary material for: Locomotor activity as an effective measure of the severity of inflammatory arthritis in a mouse model
Source: PLoS One. 2024 Jan 17;19(1):e0291399. doi: 10.1371/journal.pone.0291399 (PMC10793911; doi:10.1371/journal.pone.0291399)
Supplement: S6 Table — Comparisons of disease phases (day 8, day 14) for the two dietary groups were performed by repeated measures 2-way ANOVA. (PDF) [file pone.0291399.s006.pdf]

S6 Table. ANOVA tables for the indicated parameters. Comparisons of disease phases (day 8, day 14) for the two dietary groups were performed by repeated measures 2-way ANOVA.

**Active movements (n) / 23 h**	**F (DFn, DFd)**	**P value**
Day x Diet	F (1, 14) = 0.1884	P=0.6709
Day	F (1, 14) = 14.75	P=0.0018
Diet	F (1, 14) = 15.07	P=0.0017
**Active movements (n) / 7pm-7am**		
Day x Diet	F (1, 14) = 0.003051	P=0.9567
Day	F (1, 14) = 9.367	P=0.0085
Diet	F (1, 14) = 13.73	P=0.0024

**Horiz. Mov. (n) / 23 h**	**F (DFn, DFd)**	**P value**
Day x Diet	F (1, 14) = 0.2468	P=0.6270
Day	F (1, 14) = 7.404	P=0.0166
Diet	F (1, 14) = 10.50	P=0.0059
**Horiz. Mov. (n) / 7pm-7am**		
Day x Diet	F (1, 14) = 0.00506	P=0.9443
Day	F (1, 14) = 5.526	P=0.0339
Diet	F (1, 14) = 9.753	P=0.0075

**Rear movements (n) / 23 h**	**F (DFn, DFd)**	**P value**
Day x Diet	F (1, 14) = 2.329	P=0.1493
Day	F (1, 14) = 22.10	P=0.0003
Diet	F (1, 14) = 3.519	P=0.0817
**Rear movements (n) / 7pm-7am**		
Day x Diet	F (1, 14) = 1.601	P=0.2264
Day	F (1, 14) = 10.51	P=0.0059
Diet	F (1, 14) = 4.754	P=0.0468

**Fine movements (cm) / 23 h**	**F (DFn, DFd)**	**P value**
Day x Diet	F (1, 14) = 1.359	P=0.2633
Day	F (1, 14) = 167.9	P<0.0001
Diet	F (1, 14) = 0.2219	P=0.6449
**Fine movements (cm) / 7pm-7am**		
Day x Diet	F (1, 14) = 2.051	P=0.1741
Day	F (1, 14) = 0.2034	P=0.6589
Diet	F (1, 14) = 0.7315	P=0.4068

**Active time (min) / 23 h**	**F (DFn, DFd)**	**P value**
Day x Diet	F (1, 14) = 0.1879	P=0.6713

Day	F (1, 14) = 14.72	P=0.0018
Diet	F (1, 14) = 15.08	P=0.0017
**Active time (min) / 7pm-7am**		
Day x Diet	F (1, 14) = 0.00297	P=0.9573
Day	F (1, 14) = 9.357	P=0.0085
Diet	F (1, 14) = 13.74	P=0.0023

**Horiz. Mov. (min) / 23 h**	**F (DFn, DFd)**	**P value**
Day x Diet	F (1, 14) = 0.2517	P=0.6237
Day	F (1, 14) = 7.434	P=0.0164
Diet	F (1, 14) = 10.44	P=0.0060
**Horiz. Mov. (min) / 7pm-7am**		
Day x Diet	F (1, 14) = 0.00651	P=0.9368
Day	F (1, 14) = 5.576	P=0.0332
Diet	F (1, 14) = 9.723	P=0.0076

**Fine movements (min) / 23 h**	**F (DFn, DFd)**	**P value**
Day x Diet	F (1, 14) = 1.039	P=0.3253
Day	F (1, 14) = 2.261	P=0.1549
Diet	F (1, 14) = 0.00024	P=0.9877
**Fine movements (min) / 7pm-7am**		
Day x Diet	F (1, 14) = 1.175	P=0.2967
Day	F (1, 14) = 0.2836	P=0.6027
Diet	F (1, 14) = 0.4851	P=0.4975

**Speed (cm/s) / 23 h**	**F (DFn, DFd)**	**P value**
Day x Diet	F (1, 14) = 0.4031	P=0.5357
Day	F (1, 14) = 37.17	P<0.0001
Diet	F (1, 14) = 3.481	P=0.0832
**Speed (cm/s) / 7pm-7am**		
Day x Diet	F (1, 14) = 0.9077	P=0.3569
Day	F (1, 14) = 28.90	P<0.0001
Diet	F (1, 14) = 5.254	P=0.0379
