# Supplementary figures and images for: Locomotor activity as an effective measure of the severity of inflammatory arthritis in a mouse model
Source: PLoS One. 2024 Jan 17;19(1):e0291399. doi: 10.1371/journal.pone.0291399 (PMC10793911; doi:10.1371/journal.pone.0291399)

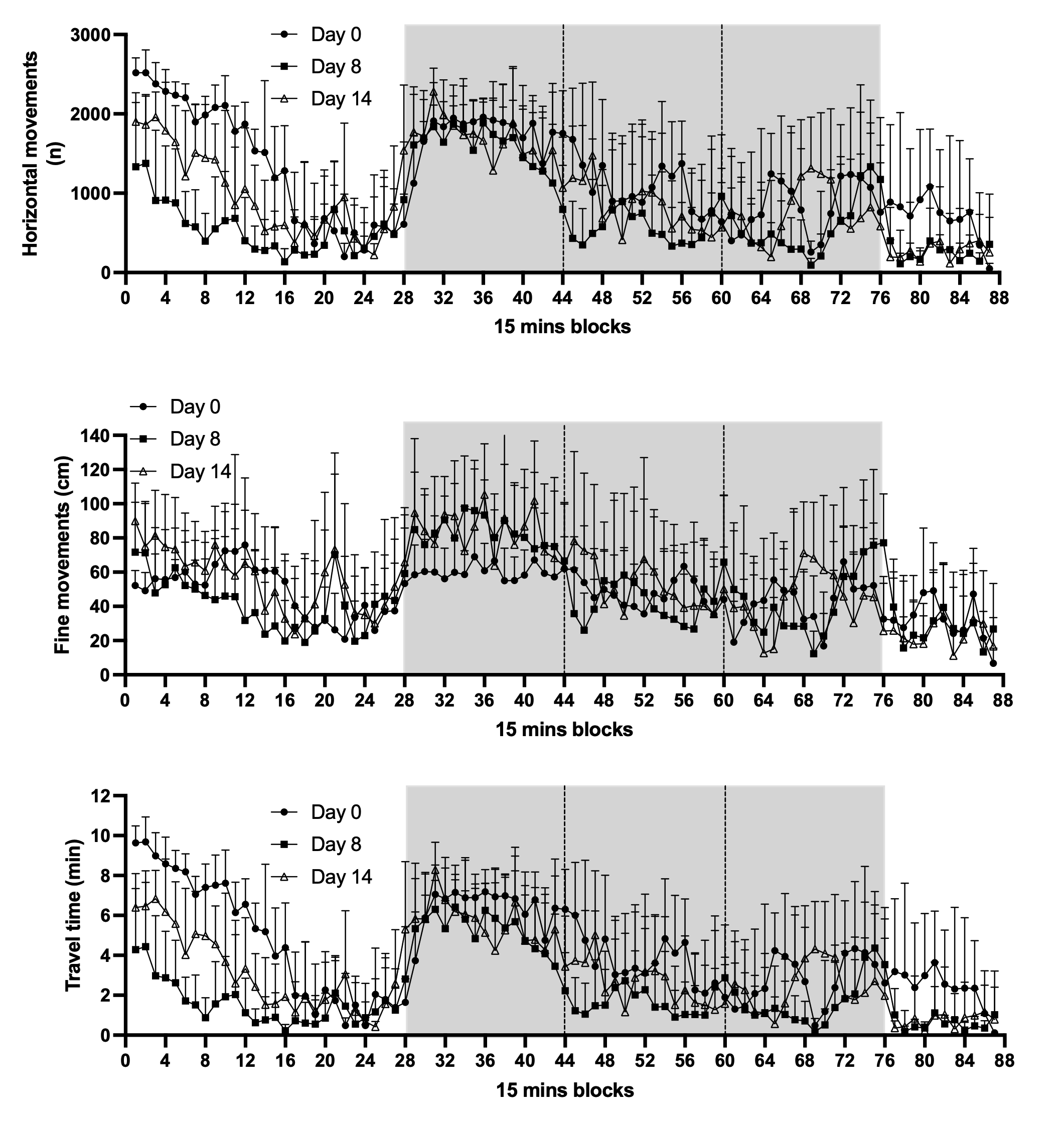

Supplement: S1 Fig — Mice were monitored with the smart cage system for 23 h on Day 0, and on Days 8 and 14 after inducing arthritis with arthritogenic K/BxN serum. (A) The number of horizontal movements, (B) the number of fine movements and (C) the travel time of the animals are shown over the 23h period divided into 15-minute blocks. Values are means +/- SD, n = 6–8 animals per group. The grey areas represent the night hours (lights out at 7pm until lights on at 7am). The night hours were divided in three blocks of 4 hours. (TIFF) [file pone.0291399.s007.tiff]

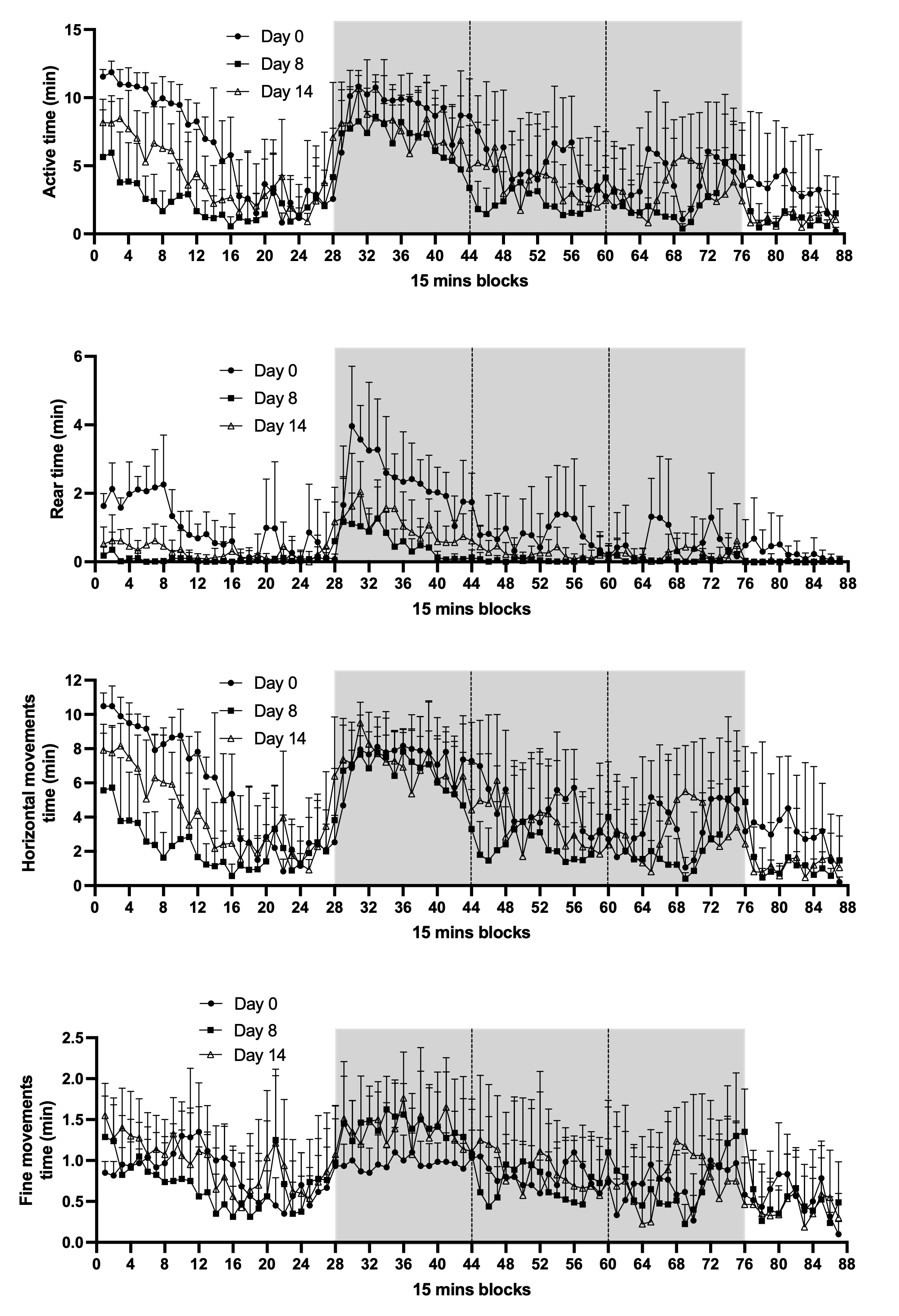

Supplement: S2 Fig — Mice were monitored with the smart cage system for 23 h on Day 0, and on Days 8 and 14 after inducing arthritis with arthritogenic K/BxN serum. (A) The active time, (B) the rearing time, (C) the horizontal movements time, and (D) the fine movements time of the animals are shown over the 23h period divided into 15-minute blocks. Values are means +/- SD, n = 6–8 animals per group. The grey areas represent the night hours (lights out at 7pm until lights on at 7am). The night hours were divided in three blocks of 4 hours. (TIFF) [file pone.0291399.s008.tiff]

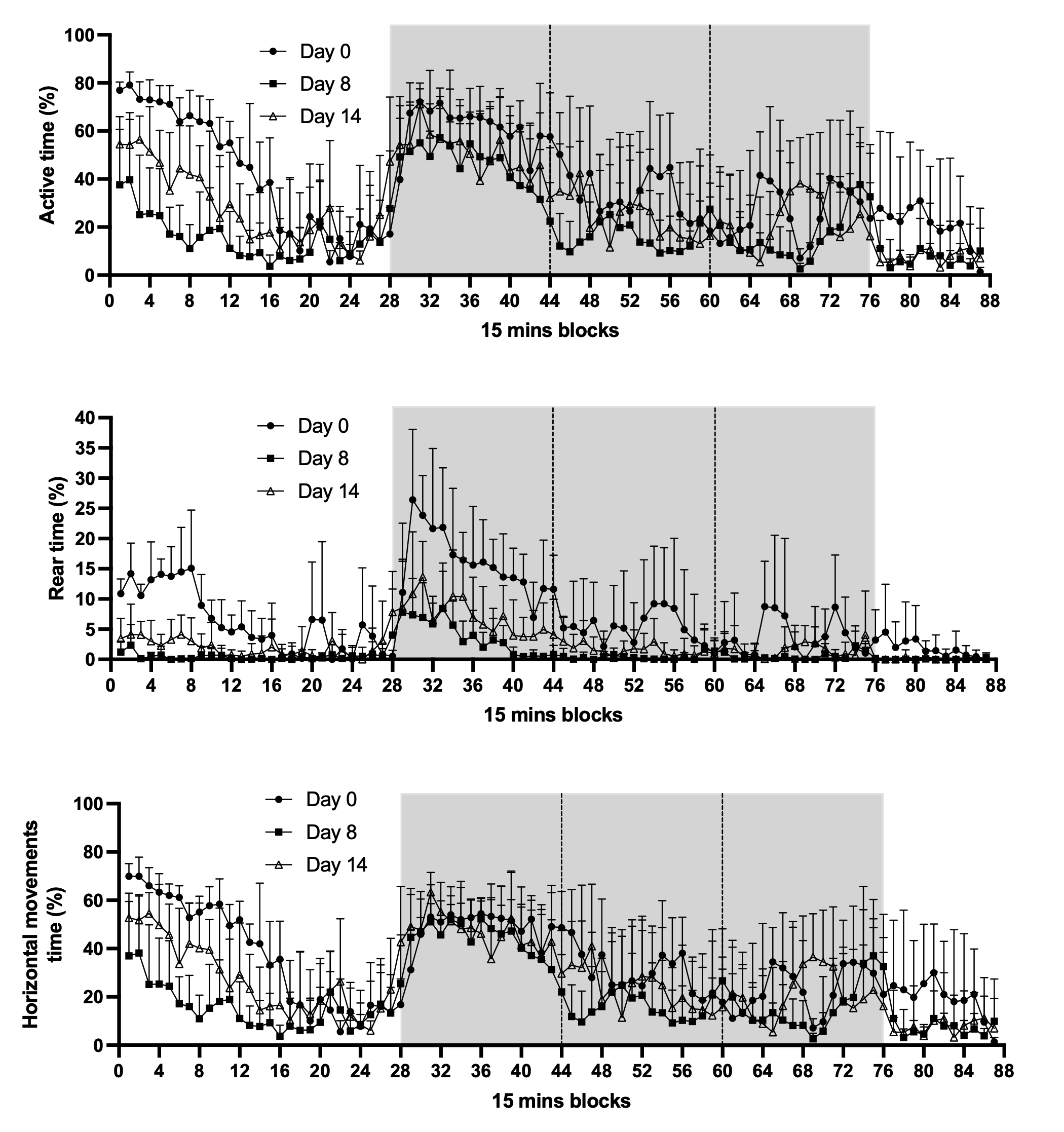

Supplement: S3 Fig — Mice were monitored with the smart cage system for 23 h on Day 0, and on Days 8 and 14 after inducing arthritis with arthritogenic K/BxN serum. (A) The percent of active time, (B) the percent of rearing time, and (C) the percent of horizontal movements time are shown over the 23h period divided into 15-minute blocks. Values are means +/- SD, n = 6–8 animals per group. The grey areas represent the night hours (lights out at 7pm until lights on at 7am). The night hours were divided in three blocks of 4 hours. (TIFF) [file pone.0291399.s009.tiff]

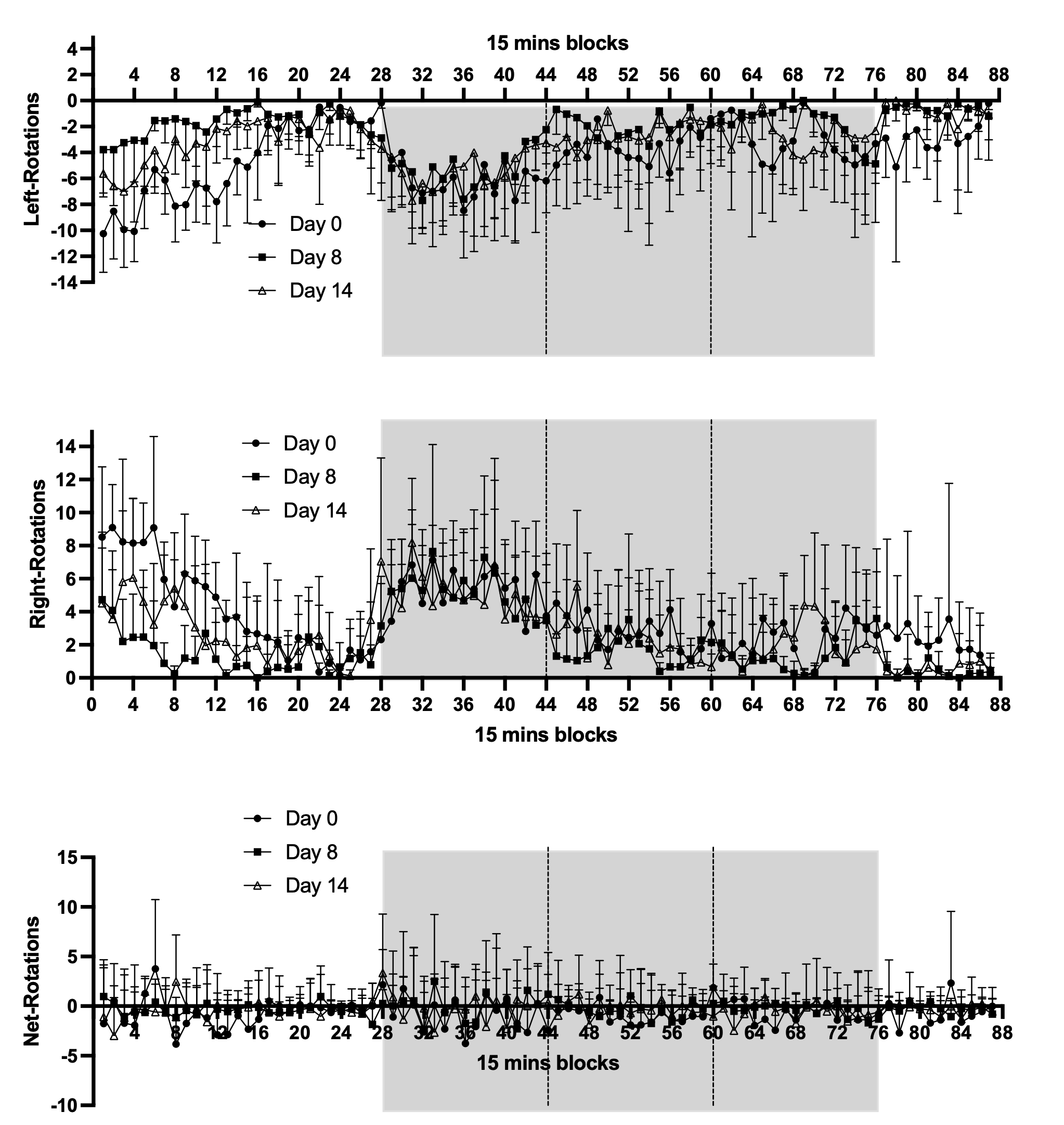

Supplement: S4 Fig — Mice were monitored with the smart cage system for 23 h on Day 0, and on Days 8 and 14 after inducing arthritis with arthritogenic K/BxN serum. (A) The number of left rotations, (B) the number of right rotations, and (C) the number of net rotations are shown over the 23h period divided into 15-minute blocks. Values are means +/- SD, n = 6–8 animals per group. The grey areas represent the night hours (lights out at 7pm until lights on at 7am). The night hours were divided in three blocks of 4 hours. (TIFF) [file pone.0291399.s010.tiff]

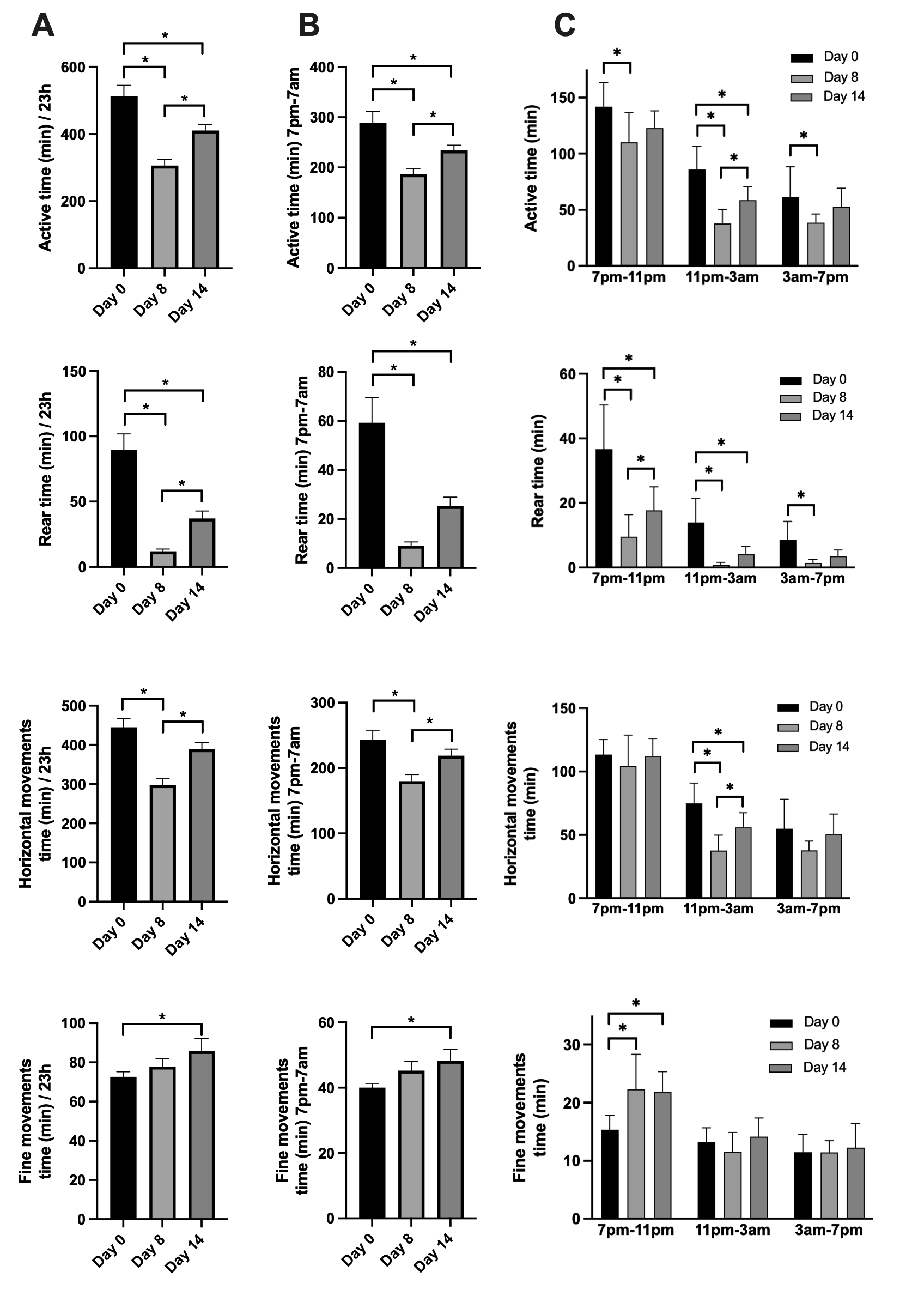

Supplement: S5 Fig — Values for the full 23 h period (A), from 7pm-7am (lights out—lights on) (B), and for the indicated 4 h blocks within the 7pm-7am period (C) are shown. Values are means +/- SD, n = 6–8 animals per group. Data in (A) and (B) were analyzed by 1-way repeated measures analyses. Data in (C) were analyzed by 2-way repeated measures analyses. All data were analysed by fitting a mixed effects model since data were missing at day 0 for two of the eight animals. See S4 Table for Mixed effects analysis tables. *Values are different as determined by the Tukey multiple comparisons test, p<0.05. (TIFF) [file pone.0291399.s011.tiff]

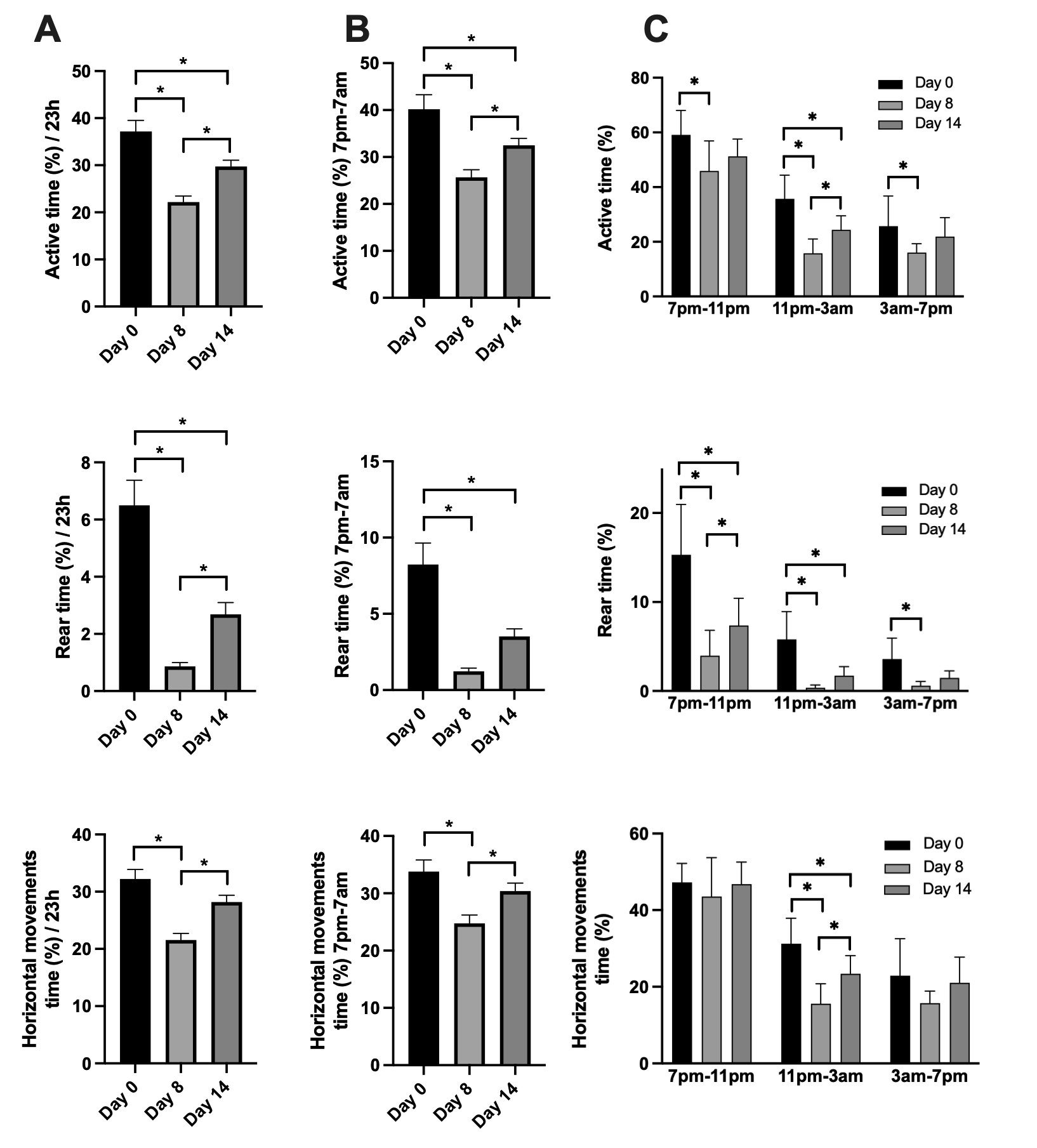

Supplement: S6 Fig — Values for the full 23 h period (A), from 7pm-7am (lights out—lights on) (B), and for the indicated 4 h blocks within the 7pm-7am period (C) are shown. Values are means +/- SD, n = 6–8 animals per group. Data in (A) and (B) were analyzed by 1-way repeated measures analyses. Data in (C) were analyzed by 2-way repeated measures analyses. All data were analysed by fitting a mixed effects model since data were missing at day 0 for two of the eight animals. See S4 Table for Mixed effects analysis tables. *Values are different as determined by the Tukey multiple comparisons test, p<0.05. (TIFF) [file pone.0291399.s012.tiff]

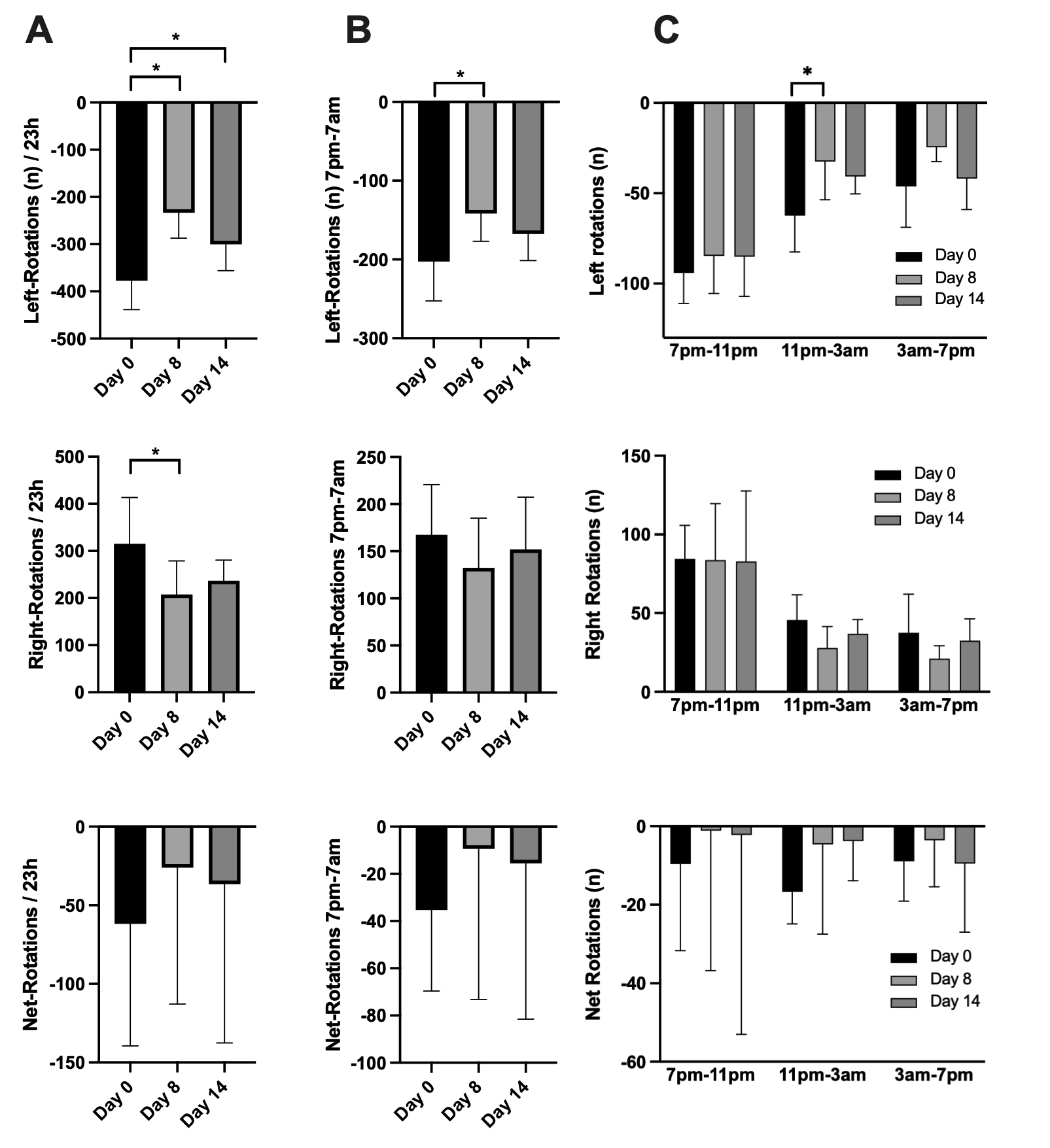

Supplement: S7 Fig — Values for the full 23 h period (A), from 7pm-7am (lights out—lights on) (B), and for the indicated 4 h blocks within the 7pm-7am period (C) are shown. Values are means +/- SD, n = 6–8 animals per group. Data in (A) and (B) were analyzed by 1-way repeated measures analyses. Data in (C) were analyzed by 2-way repeated measures analyses. All data were analysed by fitting a mixed effects model since data were missing at day 0 for two of the eight animals. See S4 Table for Mixed effects analysis tables. *Values are different as determined by the Tukey multiple comparisons test, p<0.05. (TIFF) [file pone.0291399.s013.tiff]

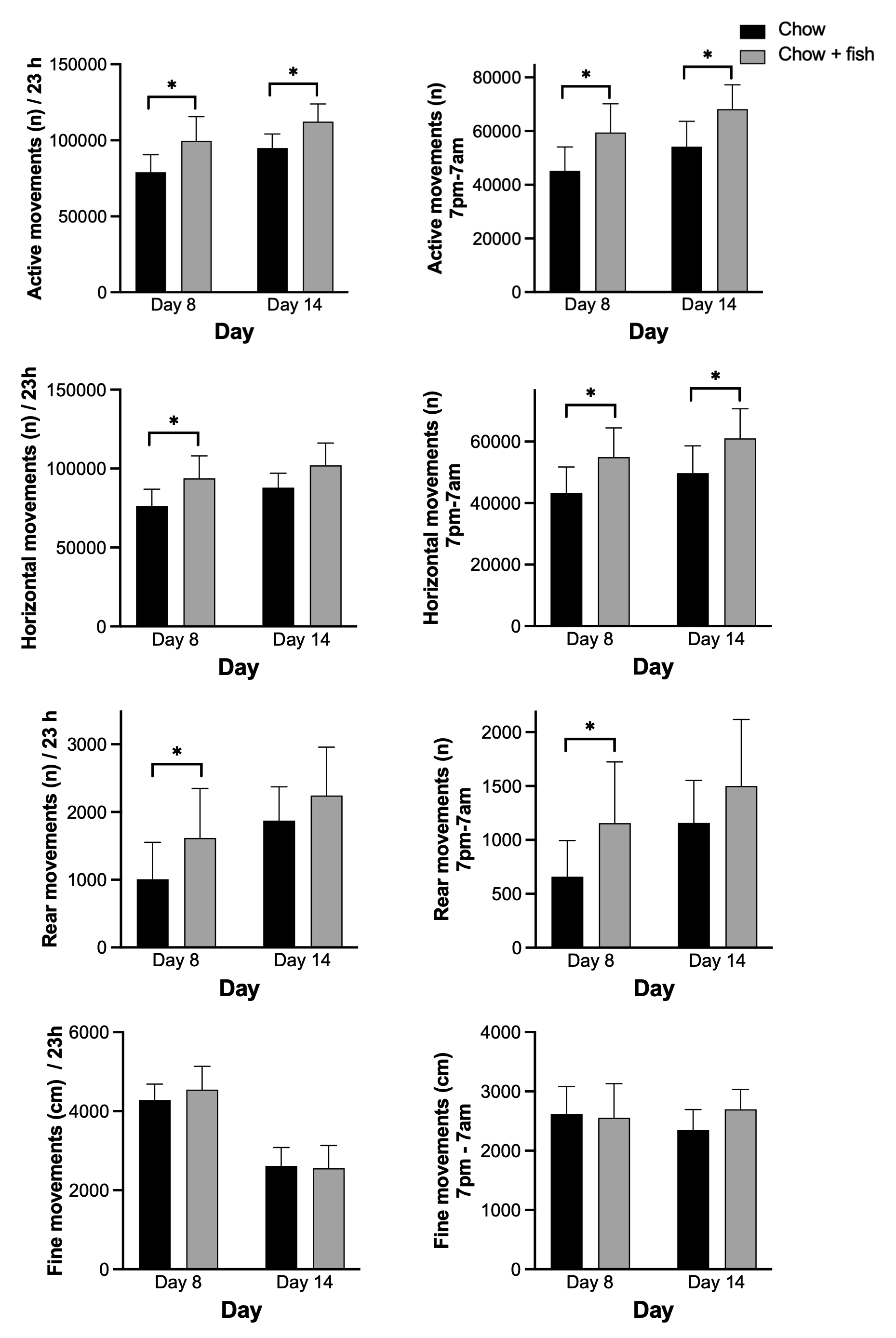

Supplement: S8 Fig — Mice consuming a chow diet, or a chow diet supplemented with fish oil were monitored with the smart cage system for 23 h on days 8 and 14 after inducing arthritis with arthritogenic K/BxN serum. Total distance travelled and the number of active movements for the full 23h period, and from 7pm-7am (lights out—lights on) are shown. Values are means +/- SD, n = 8 animals per group. * Values are different determined by two-way repeated measures ANOVA tests with Šídák’s multiple comparison analyses, p<0.05. See S6 Table for ANOVA tables. (TIFF) [file pone.0291399.s014.tiff]

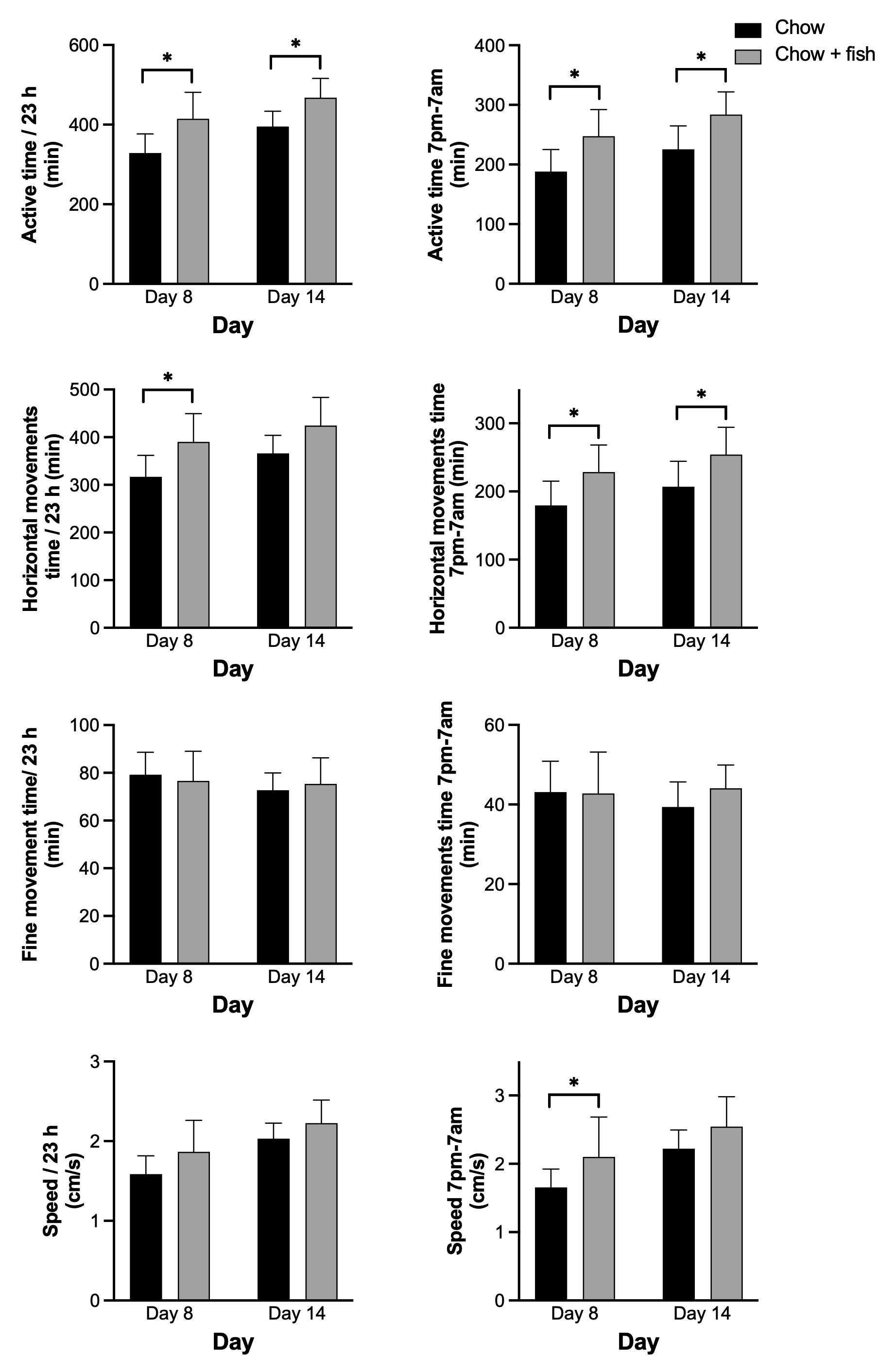

Supplement: S9 Fig — Mice consuming a chow diet, or a chow diet supplemented with fish oil were monitored with the smart cage system for 23 h on days 8 and 14 after inducing arthritis with arthritogenic K/BxN serum. Total time spend on each activity and mean speed for the full 23h period, and from 7pm-7am (lights out—lights on) are shown. Values are means +/- SD, n = 8 animals per group. * Values are different determined by two-way repeated measures ANOVA tests with Šídák’s multiple comparison analyses, p<0.05. See S6 Table for ANOVA tables. (TIFF) [file pone.0291399.s015.tiff]
